# 96 shRNAs designed for maximal coverage of HIV-1 variants

**DOI:** 10.1186/1742-4690-6-55

**Published:** 2009-06-04

**Authors:** Glen John Mcintyre, Jennifer Lynne Groneman, Yi-Hsin Yu, Angel Jaramillo, Sylvie Shen, Tanya Lynn Applegate

**Affiliations:** 1 Johnson and Johnson Research Pty Ltd, Level 4 Biomedical Building, 1 Central Avenue, Australian Technology Park, Eveleigh, NSW, 1430, Australia

## Abstract

**Background:**

The RNA interference (RNAi) pathway is a mechanism of gene-suppression with potential gene therapy applications for treating viral disease such as HIV-1. The most suitable inducer of RNAi for this application is short hairpin RNA (shRNA) although it is limited to suppressing a single target. A successful anti-HIV-1 therapy will require combinations of multiple highly active, highly conserved shRNAs to adequately counter the emergence of resistant strains.

**Results:**

We calculated the percentage conservations of 8, 846 unique 19 nucleotide HIV-1 targets amongst 37, 949 HIV-1 gene sequence fragments containing 24.8 million 19 mers. We developed a novel method of determining conservation in 'profile' sets of 5 overlapping 19 mer sequences (covering 23 nucleotides in total) to ensure that the intended conservation of each shRNA would be unaffected by possible variations in shRNA processing. Ninety six of the top ranking targets from 22 regions were selected based on conservation profiles, predicted activities, targets and specific nucleotide inclusion/exclusion criteria. We constructed 53 shRNAs with 20 bp stems and 43 shRNAs with 21 bp stems which we tested and ranked using fluorescent reporter and HIV-1 expression assays. Average suppressive activities ranged from 71 – 75%, with 65 hairpins classed as highly active (> 75% activity). Overall we found little difference in activities from minor changes in stem length (20 cf. 21), or between neighboring targets differing by a single nucleotide in start position. However, there were several exceptions which suggest that all sequences, irrespective of similarities in target site or design, may be useful candidates. We encountered technical limitations with GFP reporter assays when the target domain was long and or when the distance between the target site and fusion junction was large. Assay performance was improved by dividing large targets into several shorter domains.

**Conclusion:**

In summary, our novel selection process resulted in a large panel of highly active shRNAs spanning the HIV-1 genome, representing excellent candidates for use in multiple shRNA gene therapies. Our core selection method ensuring maximal conservation in the processed product(s) is also widely applicable to other shRNA applications.

## Background

Human Immunodeficiency Virus type I (HIV-1) is a positive strand RNA retrovirus that causes Acquired Immunodeficiency Syndrome (AIDS) resulting in the destruction of the immune system and ultimately leading to death from opportunistic infections. UNAIDS/WHO estimate that there are ~30 – 36 million people currently infected with HIV, making it one of the worst pandemic infections in history [1]. HIV is characterized by high genetic variability which, in combination with the scale and duration of the pandemic, has resulted in the emergence of many hundreds of genetically unique strains which are classified into several major groups (M, N, and O) and then further into subtypes or clades [2,3]. There is a geographical clustering for each group and subtype, with group M the main grouping distributed globally, and clade B the most common subtype found in the USA and Europe [3]. Such sequence diversity facilitates viral escape from immune surveillance as well as emergence of antiviral drug resistance, thereby posing serious challenges for the design of vaccines and antiviral therapies.

RNAi is a recently discovered phenomenon that has the potential to be exploited in Gene therapy strategies for HIV-1 (for review see [4-6]). In mammalian cells RNAi begins with a double-stranded RNA inducer that is progressively processed from its termini by RNase III type endonucleases, firstly Drosha in the nucleus followed by Dicer in the cytoplasm, to yield a short interfering RNA (siRNA) duplex [7,8]. The duplex is unwound and loaded into the RNA induced silencing complex (RISC) in a process that favors one of the 2 strands (the guide strand) based on a difference in thermodynamic stability at the ends of the duplex [9]. The most ubiquitous natural effectors of mammalian RNAi are microRNA which are small hairpin-like RNA transcripts implicated in regulation of gene expression [10,11]. The most suitable artificial RNAi inducers available for integration into current gene therapy treatments are short hairpin RNAs (shRNAs). Sharing structural similarities to natural microRNA, shRNA consists of a short single stranded RNA transcript that folds into a 'hairpin' configuration by virtue of self-complementary regions separated by a short 'loop' sequence. Several groups simultaneously developed U6 and H1 polymerase III (pol III) promoter expression systems to deliver shRNAs, exploiting their well-defined transcription start and end points [12-19]. There are now several different single shRNA forms in use. These can be generally divided into the traditional shRNAs with short (19 – 21 bp) or long (up to 29 – 30 bp) fully matched stems and several different miRNA-like variants that may incorporate bulges, mismatches and more complex loops [20-25]. With highly active molecules obtainable from all the above formats, we chose to use traditional short shRNAs in this study as they best fitted our design strategy.

The potency of individual shRNA directed to HIV-1 or its cellular receptors has now been extensively demonstrated in culture (summarized in [26,27]). However, studies examining prolonged silencing of replicating HIV-1 over time have found that the emergence of viral escape mutants occurs rapidly and can render a highly active shRNA ineffective in less than 1 month [28,29]. HIV-1 resistance to shRNA has been shown to occur through small sequence changes which alter the structure or sequence of the targeted region [29,30]. Thus, rather than circumventing the RNAi response per se, HIV-1 escape mutants have thus far only rendered individual shRNA ineffective. Mathematical modeling and related studies suggest that combinations of perhaps as few as 4 different hairpins may effectively curb the emergence of viral escape mutants [27,31,32]. Hence, there is a need for a collection of highly active, highly conserved shRNAs against HIV-1 for assembly into combinations.

There are over 170 published siRNAs and shRNAs reportedly tested against HIV-1. Two studies, by ter Brake *et. al*. (2006) and Naito *et. al*. (2007), have each contributed large sets of sequences specifically designed to be conserved in different viral strains [33,34] (Additional file 1). ter Brake *et. al*. scanned 170 complete HIV-1 genomes, irrespective of clade, in 20 nucleotide (nt.) windows and identified 19 highly conserved regions that matched at least 75% of these [34]. The authors created 86 partially overlapping shRNAs without considering predicted activities, and measured suppressive activities with a replicating HIV assay. While their shRNAs were designed with 19 bp stems, a loop sequence was used with potential to partially collapse through self complementarity, thus likely resulting in 21 bp stems [12,35-37]. Only 1 in 4 shRNAs was highly active, a figure in line with expectations of activity from randomly selected siRNAs [38-42]. In the second study, Naito *et. al*. scanned 495 near-complete HIV-1 group M genomes in 21 nt. windows and identified 216 sequences that were conserved in > 70% of strains [33,43]. Twenty three highly-conserved and 18 moderately-conserved sequences were selected with a bias towards those with high predicted activities. These sequences were synthesized as siRNAs with 21 bp duplexes plus 2 nucleotide overhangs and tested for suppressive activity by measuring degradation of a reporter mRNA via RT-PCR. Thirty nine of the 41 siRNAs tested were found to be > 60% active. However, these findings may not directly translate to shRNA studies as siRNA activity is not necessarily maintained in corresponding shRNAs.

Although most traditional shRNA design begins with a designed 19 nucleotide siRNA core, the success of siRNA to shRNA conversion is not guaranteed. There is still uncertainty surrounding shRNA processing and the precise identity of the processed siRNA products. Rigid conservation estimates used to design the shRNA stem may be invalidated if the sequence of the processed siRNA product(s) differs from that intended. This could occur if flanking and or loop sequences were incorporated during processing into the processed siRNA(s). Sequences external to the shRNA stem are not commonly designed to match the target, and hence not often considered in estimates of target conservation.

The aim of this study was to create a collection of highly active and highly conserved shRNA target sequences for HIV-1 using all available sequence information, such that intended conservation levels would be maintained in the processed siRNA product(s). We made 96 shRNAs using a novel method for selecting shRNA targets with conservation 'profiles' that consider 5 overlapping 19 nt. sequences per target, and tested their activities with fluorescent reporter and HIV expression assays.

## Results

### Nomenclature of an shRNA core design for variable shRNA processing

**Figure 1:**
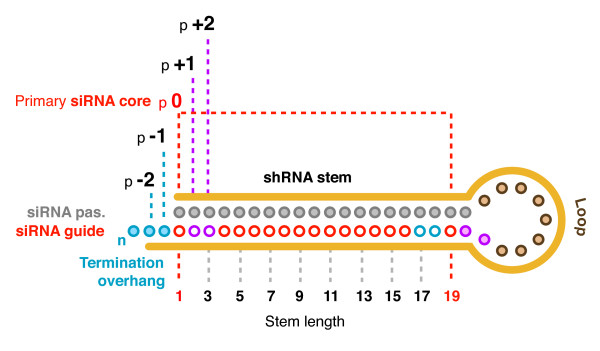
**Core placement and the surrounding nucleotides**. The shRNAs in this study were built around a designed 19 nucleotide siRNA core. We positioned the core at the base terminus of the shRNA (the open end), and extended the stem as necessary at the loop terminus (to 20 or 21 bp). The lower strand, or anti-sense strand region, was designed to be the intended siRNA guide strand which was therefore complementary to the target. Our shRNAs were designed so that the position of the first nucleotide of the primary core (p0) corresponds with the first base pair at the base terminus. The positions of the first and last nucleotides of the flanking p-2, p-1, p+1 and p+2 positions in the expected guide strand are shown (- positions in blue, + positions in purple). We considered the sequence of these flanking bases when selecting targets and estimating core conservations as it is presently unclear if these positions are incorporated in the processed siRNA product(s). The example shown is for a 20 bp stem, so that the last nucleotide of the p+2 position is also the last nucleotide of the loop. Likewise, shRNAs with 21 bp stems, the last nucleotide of the p+2 position is the last nucleotide of the stem.

We developed a novel shRNA design method to ensure that the processed siRNA product(s) retained their intended level of conservation irrespective of possible variations in shRNA processing (Figure 1). Each hairpin in this study was designed around a 19 bp siRNA target that we positioned at the base terminus or 'open' end of the shRNA shown to be the primary region responsible for suppressive activity [44]. We called the 19 mer siRNA target the 'primary core', and the first nucleotide of this core the 'p0' position. The 2 adjacent overlapping 19 mers 1 and 2 nucleotides upstream of the p0 position were referred to as the p-2 and p-1 positions, and the equivalent downstream ones were p+1 and p+2. By also considering the conservation of the surrounding sequences, our design ensures that even if shRNA processing shifts within 1 – 2 nucleotides from the expected p0 position, the resultant siRNA guide strand(s) will remain fully matched to the target.

### Assembling the HIV-1 data for conservation analysis

HIV-1 sequence data was compiled from 2 sources; publicly available sequence from the Los Alamos National Laboratory (LANL; ) and proprietary sequence information from Virco  (Additional file 2). The LANL data set included all near full-length genome sequences and gene sequence fragments as of December 2006. HIV-2 and SIV sequences were examined and excluded as they were sufficiently divergent to the NL4-3 HIV-1 reference strain [Genbank:AF324493]. The Virco data set was a small, but highly relevant private data set obtained from 105 HIV-1 infected persons from Europe. It contained only gene-specific sequences for the 6 accessory genes; Tat, Rev, Vif, Vpu, Vpr and Nef. The combined HIV-1 data set contained 24, 861, 276 separate 19 mers from 37, 949 individual partial gene sequences. These sequences spanned the 6 accessory genes, the 3 core poly-protein genes and the long terminal repeat (LTR).

### Creating the shRNA target set

We made a bioinformatic tool to compile potential 19 mer HIV-1 targets by sub-dividing the sequence of the NL4-3 strain into gene-specific sets (Additional file 2). The NL4-3 laboratory strain was chosen for a reference strain to match our reporter sequences and to evaluate the activity of all potential targets. By using individual gene sets we generated 8, 846 unique 19 mer sequences, excluding overlapping targets and LTR duplicate sequences. Due to several inter gene gaps, we omitted ~2% of potential targets, including the highly structured psi (ψ) region between the 5' LTR and Gag.

### Calculating conservations

We made another tool to determine the percentage conservation of any given 19 mer within the HIV-1 sequence sets. Conservations were calculated by sequentially comparing each 19 mer from the NL4-3 gene sets against each 19 mer in the corresponding 24.8 million HIV-1 variant gene sets in more than 44.5 billion comparisons. Only perfectly matched sequences were considered. We created a 'conservation profile' of 5 overlapping sequences for each NL4-3 19 mer core that included conservation estimates for the p0 primary core sequence, and the 2 flanking sequences on either side: p-2, p-1, p+1 and p+2. Each conservation profile was calculated in several groups: all subtypes, LANL clade B only, and accessory gene Virco sequences. The percentage conservation for the total 23 mer in each profile set was also calculated and considered. In addition to conservations, we also generated predicted activities using the Reynolds *et. al*. siRNA design rules [39], and recorded the gene or protein products for each core.

We found that 2.8% of the 19 mers were > 70% conserved across all subtypes and 0.5% were > 90% conserved. Conservations were higher for the clade B subtypes, with 14% > 70% conserved and 1.2% > 90% conserved, being derived from the LTR, Pol, Vif and Env regions. We also found 0.5% of LTR sequences were 100% conserved in all clade B subtypes. There was at least one 19 mer from each gene that was conserved in at least 80% of LANL clade B sequences, with the exceptions of Vpu, Rev and Vpr which was the most poorly conserved. The most highly conserved Vpr sequence was conserved in 57% of LANL clade B sequences, and in only 36% of sequences from all clades. We found there was a > 10% difference in conservation of the p0 and p **+- **1 positions for 7% of potential targets, and a > 10% difference in conservation of the p0 and p **+- **2 positions for 14% of potential targets.

### Core selection criteria

Target selection was based on a combination of conservations, predicted activities, target diversity and specific nucleotide inclusion and exclusion criteria in line with our described shRNA design method. The centrally located p0 19 mer was considered the most important. We typically disregarded p0 19 mers if the conservations of flanking positions were notably reduced (p +- 1 > 5% or p +- 2 > 10%), or if the total conservation of the entire 23 mer region was low. We filtered all potential 19 mers to remove those with consecutive runs of 4 or more 'T' or 'A' nucleotides to avoid the formation of pol III termination signals within the shRNA stem. We placed a bias to select sites where the initiating nucleotide at p0 was a purine ('A' or 'G') for efficient initiation of pol III transcription [45]. Where possible, we selected the primary cores so that the sequence of the first and the second nucleotides upstream of the p0 core starting position were purines to match potential 'U' remnants incorporated at the 3' end of the guide strand(s) via pol III termination.

### The chosen cores

**Figure 2:**
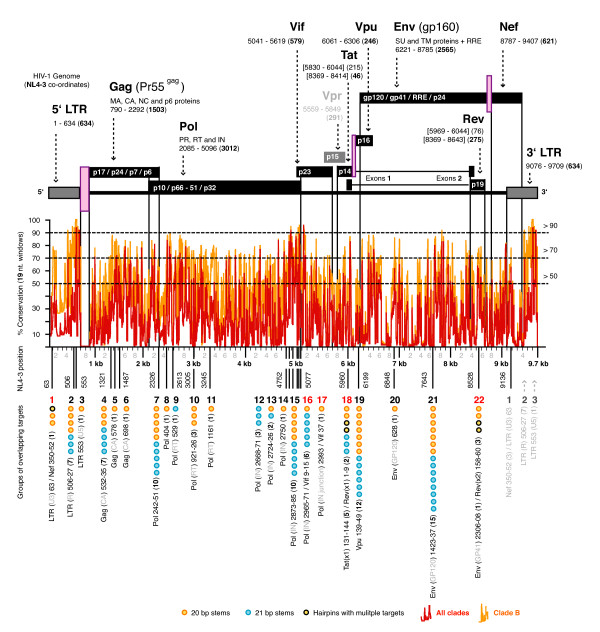
**Conservations and the 22 targeted regions**. The position and length of each gene in NL4-3 is shown above the corresponding position in the conservation graph for NL4-3. Conservations were calculated for 8, 846 19 mers from the NL4-3 gene sets against 24.8 million 19 mers in the corresponding HIV-1 variant gene sets, and plotted as conservations for all subtypes, and the clade B only subtypes. The NL4-3 genomic co-ordinates for the starting position of the 22 regions covering the 96 selected targets are given below the conservation graphs. The 22 regions and their component targets (divided into the targets made as hairpins with 20 and 21 bp stems) are shown at the bottom. Region numbers in red are the regions that have overlapping gene targets (e.g. region 1 hairpins include #1, #95 and #96, and target both the LTR and Nef). Regions 1, 2 and 3 within the LTR are repeated in light grey to indicate their overlapping target positions.

**Figure 3:**
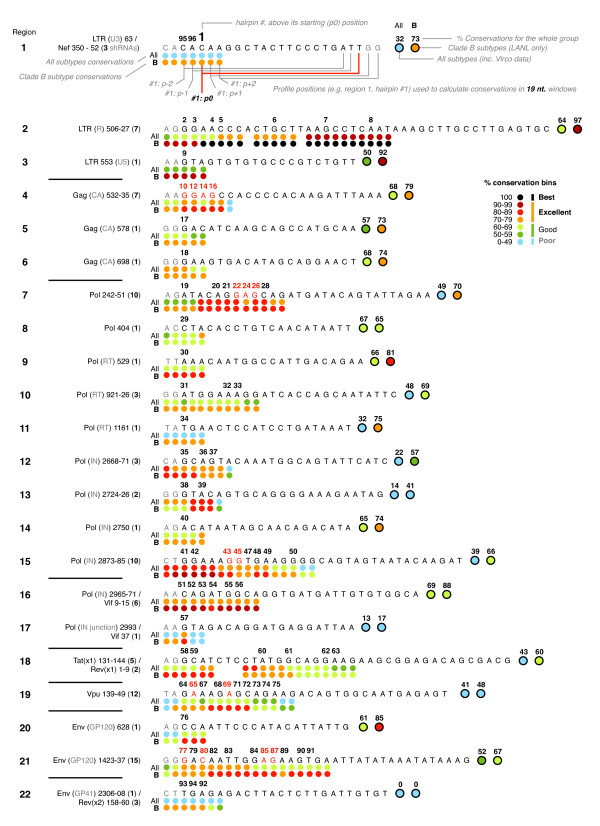
**Sequence and conservation profiles for the 96 targets**. The sequences of the 96 targets are shown in their 22 overlapping regions. The label column (left) shows the region number, the target gene, the target co-ordinates (relative to the start of the gene) and the number of shRNAs in each group. The sequence column (right) shows the shRNA # above the first nucleotide of the p0 position. shRNA #s shown in red are target start positions shared by two matched shRNAs; 20 and 21 bp stem shRNAs. The percentage conservations of 19 mer windows in all clades, and clade B subtypes only, are shown as colored-coded circles below the starting position of each 19 mer sequence within the profile sets of 5 overlapping sequences. Total conservations for each region are similarly shown at the end of each region. shRNA #s generally proceed in order, except for #95 and #96, which were selected from the Nef target set but are shown on their overlapping LTR target (region 1). An example was made of shRNA #1 with its p0 and p+-1 and p +-2 position 19 mer sequences indicated in more detail.

96 targets were selected and came from 22 distinct regions containing highly conserved sequence (Figure 2) (Additional file 2). Fourteen of these regions were unique when compared with previously published anti-HIV siRNAs and shRNAs. There was at least one core targeted to all gene fragment sets, excluding Vpr. The 96 targets consisted of 8 to the LTR, 9 to Gag, 32 to Pol, 7 to Pol/Vif (overlapping gene targets), 7 to Tat/Rev, 12 to Vpu, 16 to Env, 2 to Env/Rev, 3 to Nef/LTR (Figure 3). The average conservation profile of the 96 cores was 65 – 67% for all clades, and 78 – 80% for the LANL clade B sequences. While the average conservations of all 5 positions in our top ranking target profiles were intentionally close, there were several profiles with as much as a 70% difference in conservation between p0 and one of the flanking positions. The conservation estimates using the Virco data set were within 2% of the corresponding LANL data set across all clades. For comparison, we calculated the conservations for 100+ previously published targets and found that less than a 1/4 were greater than 70% conserved, being on average only ~34 – 45% conserved across all clades (Additional file 1).

The LTR regions were the most highly conserved with two 23 mer target profiles (#7 and #8) matched in over 90% of all clades. There were also 3 LTR targets (#2 – #4) conserved in at least 97% of LANL clade B sequences, and 4 (#5 – #8) that were 100%. Other targets that were highly conserved in LANL Clade B included 6 Vif targets (#51 – #56) and 1 Pol target (#42) matching at least 89% and 91% respectively. The chosen cores were made into 53 hairpins with 20 bp stems, and 43 hairpins with 21 bp stems. The 19 nt. cores were made into 20 and 21 bp stems by extending each primary core 1 or 2 nt. at the 3' or loop end to match the p+1 and p+2 target positions. We made 14 matched pairs: targets that had both a 20 bp and 21 bp hairpin. Where possible, the remaining sequences external to the core were selected to match the target (as detailed in Additional file 2). Synthetic oligonucleotide templates were prepared for each shRNA, and assembled in standard plasmids for expression by the human H1 promoter.

### Screening for inherent suppressive activity

The suppressive activity of each hairpin was initially screened using gene-specific fluorescent fusion reporters in a transient expression assay. Each reporter contained GFP fused upstream to one of the accessory genes, core genes or the LTR with stop codons placed between the 2 domains. Thus, each reporter produced a fused mRNA target comprised of GFP plus the HIV-1 gene from which only the GFP domain was translated. This was engineered to remove the possibility of HIV-1 protein products affecting hairpin activity. Each hairpin expression plasmid was co-transfected with 2 reporters; the corresponding target-specific GFP fusion and a non-specific AsRed-1 fusion (Clontech fluorescent proteins). Target-specific and non-specific effects on fluorescence levels were measured relative to the fluorescence levels from the plasmid backbone sample after 48 hours of hairpin and reporter expression. We have previously optimized the assay conditions to enable an approximate comparison of both target-specific suppressive activities and non-specific activities across reporters. Normalized suppressive activities were calculated by removing the overriding non-specific activity component from the apparent suppressive activity measurements.

**Figure 4:**
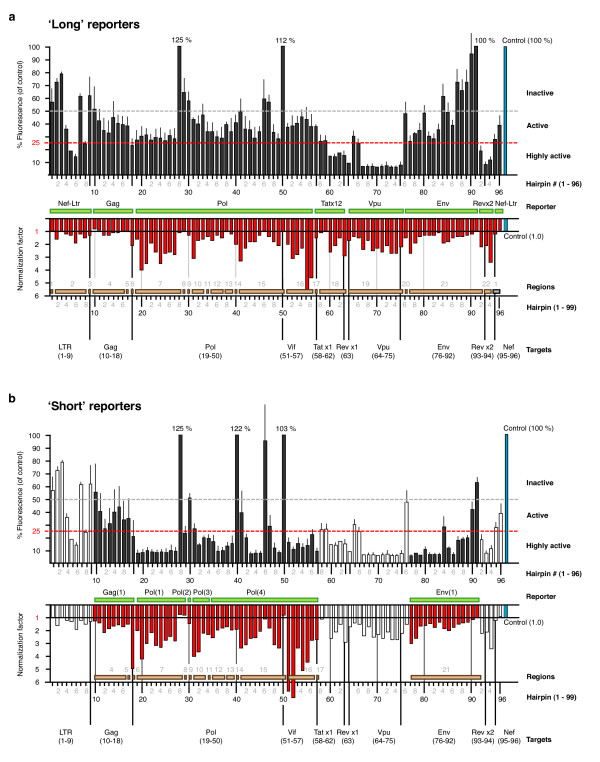
**Suppressive activities measured with fluorescent reporters**. Suppressive activities were screened using gene-specific fluorescent fusion reporters in a transient expression assay. The 96 hairpins were individually transfected with a target-specific GFP fusion reporter, and an AsRed-1 non-specific reporter. Specific activity is shown as percentage fluorescence of the unsuppressed control (black bars above; control shown in blue), and non-specific activity is shown as the fold difference (normalization factor) relative to the baseline non-specific activity of the unsuppressed control (red bars below, control shown in blue). E.g. a normalization factor of 1 is no non-specific activity, a value of 2 is twice as much non-specific activity of the baseline control (set at 1). Categorical markers divide the hairpins into inactive, active and highly active groups. The reporter used for each hairpin (green bars), and the 22 targeted regions (brown bars) are also shown. (**a**) Suppressive activities measured with the first round 'long' (core genes) and 'short' (accessory genes) reporters. (**b**) The previous long reporters were replaced with a series of shorter fragment reporters and suppressive activities were remeasured. The suppressive activities form the previous short accessory gene reporters are included for comparison (hollow bars).

The average suppressive activity across the 96 hairpins was 63%; i.e. the presence of the shRNA reduced the average level of fluorescence to 37% of the unsuppressed control (Figure 4a). Twenty two hairpins were highly active (> 75% suppressive activity), 56 were active (between 50 and 75% activity) and 18 were inactive (< 50% activity). Non-specific activities varied widely and mostly enhanced the fluorescence levels, but did not appear to correlate to suppressive activity. While the mechanism and significance of non-specific signal enhancement is not known, it is a phenomenon that we have commonly observed and have previously determined to be sequence-specific, dose-dependent and highly reproducible [44].

### Reporter length and the distance between the target site and the fusion junction can affect apparent suppressive activity

**Figure 5:**
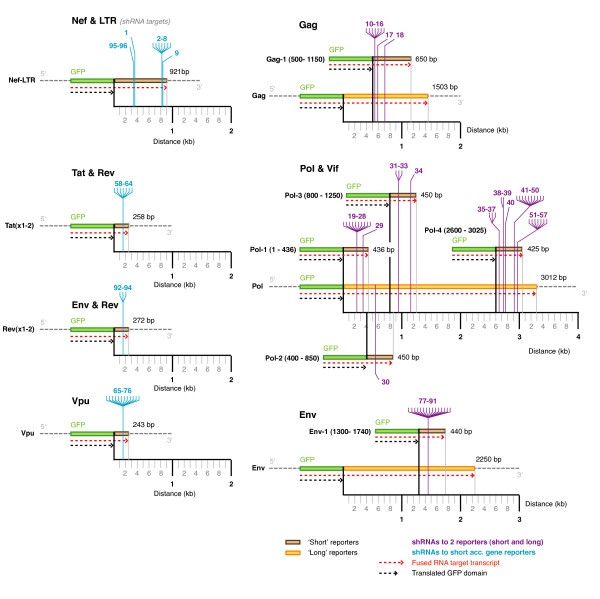
**shRNA target sites mapped to the 13 fluorescent reporters**. The target sites of the 96 shRNAs were mapped to the 13 fluorescent reporters. The 4 short accessory genes and LTR reporters are shown down the left, and the 3 long core gene reporters are shown down the right. Mapped onto the long core gene reporters were the corresponding 6 short gene fragment reporters made to retest the core gene shRNAs. Lengths are to scale to show the relative distances between the target sites, fusion junctions and total reporter lengths. n.b. some shRNAs were assayed with a different reporter to that of the gene targeted due to overlapping target sequences, e.g. the Vif matched shRNAs #51 – 57 were assayed with Pol reporters.

While we observed an expected spread of suppressive activities, the average level for the shRNAs measured with the core-gene reporters was generally lower than that observed for the shRNAs measured with the accessory-gene reporters. We observed an average percentage fluorescence of 38% for Gag, 42% Pol, 52% Env reporters vs. 46% Nef-LTR, 19% Tat exons 1 and 2, 10% Vpu and 13% Rev exon 2 reporters. We also noted that the target domains for the core-gene reporters were longer than the accessory gene reporters. The lengths of the target domains ranged from 1, 503 – 3, 012 bp for the core gene reporters but only 243 – 921 bp for the accessory genes. The shRNA target sites in the long-core reporters were also generally further away from the fusion junction between the GFP and gene-target domains. A second set of shorter core-gene reporters were constructed to determine if the length of the target domain and or the distance of the target site from the fusion junction was affecting apparent suppressive activities (Figure 5). The length of these shortened reporters ranged from 425 – 650 bp and all matching hairpins were re-assayed.

**Figure 6:**
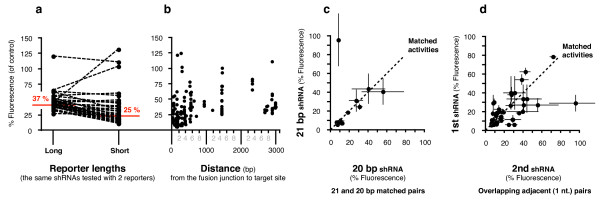
**Reporter length, target distance, matched and overlapping hairpin pairs**. (**a**) The suppressive activities of hairpins that were assayed with both long core gene reporters and shorter core gene fragment reporters, were plotted and grouped according to reporter length. (**b**) Suppressive activity was plotted against the distance of the target site from the fusion junction for all hairpins tested. (**c**) The suppressive activities of 14 matched pairs of hairpins with 20 and 21 bp stems designed to the same target sites. (**d**) The suppressive activities of 73 pairs of neighboring hairpins with target sites a single nucleotide apart.

There was a noticeable shift in the activities of most hairpins when assayed with both long and short reporters (Figure 4b). The majority of hairpins were more active when assayed with the short reporters, with the average percentage fluorescence ranging from 18 – 51% (combined average of 29%) compared to 39 – 58% (44% combined) with the longer reporters. The percentage fluorescence across the 96 hairpins including the shorter reporters was improved from an average of 37% to 25%. Sixty five hairpins were now regarded as highly active (*cf*. 22), 19 were active (*cf*. 55) and only 12 were inactive (*cf*. 19). As the activity data was not normally distributed, we performed a Wilcoxon signed rank test, the non-parametric equivalent of a paired t-test, to statistically compare the activities of the shRNAs from the matched long and short reporters (Figure 6a). This confirmed that the change in activity was due to a change in reporter length (P < 0.0001). We also plotted the suppressive activity against the distance of the target site from the fusion junction for all hairpins tested (Figure 6b). A non-parametric analysis of this data showed that there was a significant correlation between the apparent activity and distance of the target site from the fusion junction (Spearman correlation co-efficient of 0.53). However, even though there was a significant correlation, the large scatter suggests that there may be other compounding factors such as alternate secondary structure formations and changes in target-site accessibility.

### Comparing matched pairs with 20 and 21 bp stems

The 96 hairpins were comprised of 53 hairpins with 20 bp stems, and 43 hairpins with 21 bp stems. The average percentage fluorescence for the hairpins with 20 bp stems was 31%, with 30 scored as highly active. The average percentage fluorescence for the 43 hairpins with 21 bp stems was 18%, with 35 scored as highly active. As the hairpins in each group were selected individually, and hence were mostly unmatched, it was not possible to determine from this analysis if either length was necessarily more active. There were, however, 14 matched pairs of hairpins with 20 and 21 bp stems designed to the same target site. A statistical analysis of these with a Wilcoxon signed rank test showed that there was no overall significant differences in activity (p = 0.8077), although a few pairs, #45 | #46, #80 | #81, #85 | #86, exhibited notable changes (Figure 6c).

### Comparing hairpins with adjacent, overlapping targets

Seventy three adjacent pairs of hairpins had starting positions that differed by 1 nucleotide. There was a > 10% change in activity for more than a quarter of the pairs which had an average change in activity of 32%. Forty-two of the 73 pairs had the same stem length which enabled us to compare their activities with a Wilcoxon signed rank test showing that there was no significant difference (p = 0.1077) (Figure 6d). Pairing was effective in accounting for variability indicating that in most cases, activity for the 2 neighboring sites were similar (r = 0.59, p < 0.0001, one-tailed test). However, there were some notable exceptions such as the pairs #26 | #28 and #44 | #47 for which the 1 base difference had a major impact on activity.

### Screening for anti-HIV-1 activity

**Figure 7:**
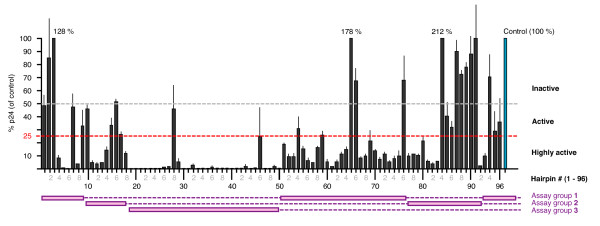
**Impact on HIV-1 expression**. Anti-HIV-1 suppressive activity was tested by transfecting each shRNA expression plasmid with the NL4-3 expression plasmid. Activities were measured as a reduction in, and expressed as percentage of, p24 production (measured as pg/ml) relative to p24 production from the unsuppressed control (shown in blue). The 96 shRNAs were measured in 3 sets (as indicated in purple) with 2 or more replicate experiments in each set (accessory gene shRNAs in one set, Gag plus Env shRNAs together, and Pol shRNAs separately).

All shRNAs were also tested in the NL4-3 HIV-1 expression assay. In this assay, shRNA expression plasmids were co-transfected with the pNL4-3 HIV-1 expression plasmid and total p24 levels were measured 2 days later. Activity was calculated as a relative percentage of the baseline p24 levels present in the plasmid backbone sample. In this assay, the average percentage p24 for the 96 hairpins was 29% of the unsuppressed control. Sixty five were classed as highly active, 16 were active and 15 were inactive (Figure 7). These groupings closely matched those from the gene fusion assay using the shorter reporters, with several exceptions. These exceptions could be due to differences in target structure and accessibility to the target site, or to influences from non-specific activities which were not distinguishable in the current format of this assay. It should also be noted that the suppressive activities for the highly active hairpins in the NL4-3 assay generally appeared greater than those observed in the gene fusion assay. However, we consider that this was likely due to differences in assay dynamics such as different shRNA to target ratios, target production times, etc. Importantly, the overall categorical and relative ranking of hairpins based on suppressive activities were similar in both assays.

### Short-listing the best sequences

We created a scoring method to rank the 96 hairpins for overall performance based on their suppressive activities, non-specific activities and percentage conservations. Each parameter was weighted approximately equally and awarded a score out of 100. Suppressive activities from both assays were tallied as 100 minus the percentage fluorescence value or percentage p24 graphed; e.g. a graphed value of 14% fluorescence scored an 86. Non-specific activity was awarded a top score of 100 for no activity if the shRNA had a normalization factor of 1.0. This was reduced accordingly for increased non-specific activity, e.g. a normalization factor of 1.2 (0.2 fold enhancement) or 0.8 (0.2 fold reduction) was only awarded 80, and so on. The percentage conservations were also tallied using the p0 position. According to this method, the 10 overall best performing hairpins from this study were: LTR 510-21 (#5), LTR 527-21 (#8), LTR 509-20 (#4), Env 1428-21 (#84), Tat 131-20 (#58), Tat 132-21 (#59), Env 1425-21 (#82), Env 1426-21 (#83), LTR 516-21 (#6), and Gag 532-21 (#11) (Additional file 2). For all 10 hairpins the percentage conservations for the p +- 1 and 2 positions were within 8% of that for the p0 position. This method of ranking was only one of many available, and a bias towards activity, or conservations may be more suitable for other applications.

## Discussion

The aim of this study was to create a collection of hairpins against HIV-1 that are suitable for potential inclusion in multiple hairpin gene therapies. We developed an original method to analyze conservations for each potential 19 nucleotide target and derive 'conservation profiles' to maximize the likelihood of maintaining conservation of the processed siRNA product(s) in the absence of detailed mechanistic information regarding shRNA processing. We selected 96 of some of the theoretical best targets, assembled them into shRNAs consistent with our design method, and characterized their activities using fluorescent reporter and replicating HIV-1 assays. The hairpins were ranked and shortlisted to highlight those potentially most suitable for inclusion in multiple shRNA gene therapies.

The relevant previous studies have used only 170 – 495 full-length HIV-1 genome sequences to estimate conservation, or the equivalent of 1530 – 4455 sequences if subdivided into individual gene sequences; just 4–11% of the sequences considered here [33,34]. In this study we examined close to 38 thousand partial sequences which included sequence information from the full-length genome data set fragmented into separate genes. There are, however, some limitations with the partial sequence set, and the LANL data in general. Some of the partial gene sequences are likely duplicate entries of sequence from the same patient, which may skew conservation estimates. However, we have compared all sequences within each gene set and found that on average 82% of sequences were unique. Therefore, our analysis contained ~7 – 20 times the number of sequences used in previous studies. We compared our conservation estimates for a set of 100+ previously published sequences against those estimated by others, and found that while many conservations were similar, some differed widely (Additional file 1) [33].

Secondly, the sequences in the LANL and Virco databases were biased towards clade B sequences from studies in the US and Europe with an average of 45% of the sequences in each being clade B. Unfortunately the greatest diversity of Group M sequences is likely to be found in places like the Democratic republic of Congo which do not have the resources for large-scale sequencing. However, at this stage of gene therapy development, clade B sequences are still the most relevant as trial sites, access to infrastructure and the initial cost of treatment will likely restrict early gene therapy to the US, Europe and other developed regions. The Virco data set was generated to represent a broad cross section of HIV-1 patients likely to be included in future European gene therapy trials. For this reason, approximately half of these patients were selected because they had been infected with Clade B.

The suppressive activities of our hairpins were first tested with a fluorescent reporter assay, followed by a more biologically relevant HIV-1 expression assay. The fluorescent reporter assay has higher throughput and can estimate inherent suppressive activity whilst providing an indication of non-specific activity. Fluorescent reporter systems are now widely used for estimating suppressive activity. However, here we show that their use is not always straightforward. For reporters with the target domain fused downstream of the fluorescent domain, we found that the length of the reporter and or the distance of the target site from the fusion junction can affect the apparent suppressive activity. This may be due to a delay between the cleavage event and complete target degradation permitting some translation. Alternate secondary structure formations and changes in target-site accessibility are also likely to be contributing factors as shown in many previous reports [30,46-51]. We do not expect that all hairpins, if measured using both a long and short reporter, will show a change in activity for a change in distance. If a hairpin was entirely inactive in a short reporter we would not expect its activity level to improve with a change in reporter length. Similarly an extremely active hairpin may cleave its target with such high efficiency that even moderate changes in reporter length or distance from the fusion junction may be inapparent. We believe hairpins with intermediate suppressive activities of ~10 – 90% will be most sensitive to changes in reporter length. This issue warrants further and specific investigation, especially given the widespread use of fluorescent reporter assays.

When we examined 20 and 21 bp stems that shared the same core start site, but differed by 1 nt. at their 3' ends, we found that overall a single nucleotide change in stem length had no statistically significant effect on suppressive activity. We were interested in comparing these activities since we had previously found that hairpins with 20 bp stems may be processed into fewer products than the more common 21 bp hairpins [44]. Our current study demonstrates that hairpins with 20 bp and 21 bp stems sharing a common core start site are frequently equally effective suppressors. With no change in the core start site, the primary siRNA product(s) may be unchanged. However, this may only be a general rule as in some instances we did note differences in the activities of the 2 stem lengths, affirming our need to better understand the details of shRNA processing.

We also compared the activities of 2 overlapping hairpins with target sites shifted by a single nucleotide, and for hairpin pairs with the same stem length found no overall significant difference in activities. However, there were a number of pairs with markedly different suppressive activities which is an observation that has also been noted by others. One study reported 2 independent series of 3 overlapping 19 bp shRNA targets differing by a single nt each, where the 1st and 3rd shRNAs were active, but the intervening one was not [34]. This is in line with our current understanding of shRNA processing, where short hairpins are believed to be processed from the 5' open end or base terminus, and of siRNA activity which is defined by specific nucleotide positioning relative to the ends [39,52-54]. Even the smallest change in core start site may be changing the siRNA(s) produced. For routine application, designing shRNA for 2 neighboring sites is probably unnecessary, although with the noted exceptions it may be worth testing the alternative n +- 1 sites if the n site has low activity, or if the region has a particularly useful attribute such as high conservation.

The standard approach to finding suitable shRNAs for HIV-1 suppression includes screening for suppressive activity and or basing conservation estimates solely on the designed core. But with the current gaps in our knowledge of shRNA processing, such screens may select for shRNAs that although active, may not be yielding the intended siRNA(s). This is because ordinary shRNA designs generally do not consider the potential contribution of surrounding sequence. Hence, the conservation for the actual processed siRNA(s) may differ from the expected processed siRNA and not maintain activity on all viral subtypes as predicted. Although we found that a large number of our 8846 potential 19 mer target profiles were equally conserved across all 5 positions, there were a significant number that differed by 10% or more between the primary and p +- 1 (7%) and p +- 2 (14%) positions. Our method reduced the variation in conservation within each of our top ranking profiles to an average of 3%. Thus, all of the selected sequences will remain highly conserved, irrespective of processing discrepancies and the precise sequence of the processed siRNA product(s). Moreover, there is no foreseeable disadvantage in applying our method now, even if it should turn out that shRNA processing generates a single siRNA product identical to of the intended core.

The results of this study will be of widespread use to others, especially since there is unrestricted use of all the identified hairpin sequences. In the absence of a detailed understanding of shRNA processing mechanisms our target selection and shRNA design methods ensure that the resulting product(s) remain conserved as intended. Moreover, the method is likely adaptable to other viral diseases with highly variable sequence. Our results regarding the use of long fluorescent reporters, as well as the changes in suppressive activity associated with target proximity and minor changes in stem length add to our basic understanding and applied use of shRNA. The collections of hairpins, both the entire theoretical set and those we selected as most suitable, are valuable resources for others working towards an RNAi therapy for HIV-1.

## Methods

### Sequence analysis tools

The tools for subdividing NL4-3 and the HIV-1 variants into 19 mers, calculating conservations, filtering sequences, and all other sequence manipulations were written in Visual Basic using Excel as an interface (Microsoft Excel X for Mac, 2001).

### Constructing 96 shRNA expression plasmids

The inserts for all but 3 of the shRNA expression plasmids were built from a single synthetic oligonucleotide ~72 – 75 nt. long (Additional file 2). Each oligonucleotide template consisted of a partial restriction enzyme recognition site (*Bam *HI), the hairpin sense (or upper) sequence, a loop sequence, the hairpin anti-sense sequence, a pol III terminator sequence, and a second partial restriction enzyme recognition site (*Hind *III). A short primer sequence (12 nt.) common to the 3' end of all oligonucleotides was also designed, annealed to each oligonucleotide template and extended with Phi-29 DNA polymerase in a single-step isothermal extension reaction to make double-stranded synthetic inserts that were then digested to create 'sticky ends' and cloned as per standard procedures (as previously described) [35]. The remaining 3 shRNA templates were created from standard complementary oligonucleotide pairs with offset ends as each shRNA core sequence contained an internal *Hind *III site making it incompatible with the Phi-29 extension method. Each insert was placed into a human H1 (pol III) expression plasmid derived from pSilencer (Ambion). All shRNA plasmids were propagated in GT116 *E. coli *cells; a cell line specifically developed for the replication of hairpin containing plasmids and vectors (Invivogen). DNA was extracted (Hi-speed Maxi-prep Kit, Qiagen), quantitated in triplicate (Nanodrop) and was sequence confirmed, using a modified protocol where necessary that enabled automated sequencing of shRNA expression plasmids possessing reaction-inhibiting secondary structure [35].

### Reporter plasmid construction

The fluorescent protein-target fusion reporter plasmids were constructed using EGFP (from pd4-d4EGFP-N1, BD Biosciences), AsRed1 (from pAsRed1-C1, BD Biosciences) and HIV-1 sequences PCR amplified from variant NL4-3 [Genbank:AF324493] (Additional file 2). Each reporter contained the fluorescent domain fused upstream of one of the accessory genes, core genes or the LTR with stop codons placed between the 2 domains to ensure that only the fluorescent protein domain was translated.

### Fluorescent reporter assay

Each shRNA expression plasmid was co-transfected with two reporters; the corresponding target-specific GFP fusion and a non-specific AsRed-1 fusion. The non-specific reporter was typically an AsRed-1-Vpr fusion, since we selected no Vpr shRNA targets. HEK293a cells (sourced from the American Type Culture Collection) were seeded at a density of 5 × 10^5 ^cells per well (6 well plates; 2 ml of medium). Cells were transfected 1 day later using 1 μg of total DNA (400 ng of shRNA expression plasmid, 300 ng of target plasmid and 300 ng of control plasmid) with 4 μl of Lipofectamine 2000 (Invitrogen) in OptiMEM (Invitrogen) to a total volume of 100 μl/well. Cells were analyzed by flow cytometry ~48 hours later (FACsCalibur and CellQuest Pro for Mac OS X, BD Biosciences). Target-specific suppression was measured as a decrease in green fluorescence (FL1 channel) and non-specific effects were measured as a change in red fluorescence (FL2 channel). The Fluorescence Index (FI) of cells in each channel was calculated by multiplying the geo mean of fluorescence by the percentage of cells that were fluorescent (only those cells gated above background). The FI of FL1 (green, target-specific activity) was normalized to remove non-specific effects (FI of FL1 normalized to the FI of FL2) and was expressed as a percentage of the FI of cells transfected only with the control plasmid that expressed no shRNA. The normalization factor was shown as a relative measure of non-specific shRNA activity (a value of 1 equals no non-specific activity, i.e. the measured non-specific activity was identical to that measured for the control plasmid that expressed no shRNA). Each sample was analyzed in triplicate, and each experiment was repeated at least 3 times with 95% confidence intervals shown (calculated from a minimum of 9 points). Every experiment included a mock transfection (i.e. no DNA) and an off-target shRNA control (to verify that on-target shRNA suppression was sequence-specific) (Additional file 2); both of which behaved as expected, and both of which were omitted from the graphs for clarity.

### HIV-1 expression assay

HEK293a cells were seeded at a density of 2 × 10^5 ^cells/well (12 well plates; 1 ml of DMEM-10). Cells were transfected ~24 hours later using 110 – 130 ng of shRNA expression plasmid (equal molar amounts) and 800 ng (3× molar amount of expression plasmid) of pNL4-3 reporter plasmid with Lipofectamine 2000 at a ratio of 1: 4 (μg DNA: μl Lipofectamine 2000) in OptiMEM to a total volume of 200 μl/well. Medium was replaced with an equal volume ~24 hours post-transfection and the cells were harvested a further ~24 hours later by centrifugation at 400 g for 10 min. at room temperature. Samples were stored at -20 °C until assayed for p24 levels (a capsid protein required for HIV-1 virion production) via Enzyme-Linked Immunosorbent Assay (ELISA) using the INNOTEST HIV antigen mAb kit (Innogenetics). The suppressive activity of each shRNA was measured as a reduction in, and expressed as percentage of, p24 production (measured as pg/ml) relative to p24 production from cells transfected with the corresponding empty expression control plasmid. The 96 shRNAs were measured in 3 sets with 2 or more replicate experiments in each set (accessory gene shRNAs in one set, Gag plus Env shRNAs together, and Pol shRNAs separately), with 95% confidence intervals shown. The values from each experiment were adjusted relative to minor changes in an internal control shRNA common to all experiments to allow comparison of the different shRNAs across experiments (#61, Tat143-21).

### Statistical analysis

All error bars on activity graphs were 95% confidence intervals (CI) (Microsoft Excel X for Mac, 2001). P values were determined as individually described using Graphpad Prism 4.0a for Mac OS X, 2003.

### Figure preparation

Figures were prepared by 

## Competing interests

This work was done by employees of Johnson and Johnson Research (JJR), for JJR.

## Authors' contributions

GJM and TLA conceived the experiments. GJM, JLG, and YHY constructed the plasmids and performed the fluorescent reporter assays. AJ and SS performed the HIV-1 expression assays. GJM, JLG and TLA analyzed and interpreted the results. GJM wrote the manuscript.

## Supplementary Material

Additional file 1 **Previously published sequences tabulated and identified by Naito *et. al*. (2007) **[33]**, were scored for conservation using our tool and compared against original conservation estimates.** Click here for file

Additional file 2 **An additional data file containing (i) a table of the the number of sequences in the HIV-1 partial gene fragments data set, (ii) a table summarizing the number of 19 nt. sequences analyzed, (iii) a summary table of the numbers of most highly conserved targets, (iv) a list of the 96 selected targets and surrounding sequence, (v) a table of the 22 overlapping regions containing the 96 targets, (vi) a list of the 20 and 21 bp matched targets, (vii) a list of the overlapping hairpins with adjacent start sites 1 nt. apart, (viii) a detailed list of the % conservation for each position in the 5 sequence profile for all 96 selected targets, (ix) a table summarizing the differences in the fluorescent reporters used, (x) a list of the hairpins in scored and ranked order, (xi) additional method information such as oligonucleotide sequences, and (xii) the NL4-3 gene sequences used to generate the targets and make the reporters.** Click here for file
