# Supplementary material for: 96 shRNAs designed for maximal coverage of HIV-1 variants
Source: Retrovirology. 2009 Jun 4;6:55. doi: 10.1186/1742-4690-6-55 (PMC2698899; doi:10.1186/1742-4690-6-55)
Supplement: Additional file 1 — Previously published sequences tabulated and identified by Naito et. al. (2007) [33], were scored for conservation using our tool and compared against original conservation estimates. [file 1742-4690-6-55-S1.pdf]

Other sequences

Conservation estimates of previously studied si- and shRNAs

One of the two most relevant prior studies compiled **129** previously published si- and shRNAs, and calculated their percentage conservations using 21 nt. windows. For targets longer than 21 nt., they evaluated all possible 21 mers and scored the most highly conserved. Their method differs from ours, in that we considered 19 nt. windows, and generally considered the base position, or first 19 mer the most relevant. To compare our method and data sets with theirs, we took the same sequences, added their **216** proposed sequences, and re-calculated conservations. We found both similarities and differences, but overall both methods agreed. The following table contains the sequence ID # (**S**), the HIV target region, the sequence, the % conservation for the first 21 nucleotides estimated by Naito *et. al.* (2007), the % conservation for the entire sequence and the first 19 nucleotides in all subtypes, % conservation for the entire sequence and the first 19 nucleotides in all clade B subtypes, and the reference. *n.b. Much of the information in the following table was reproduced from information originally compiled by* ***Naito et. al. (2007).***

				All clades		Clade B		
**S**	**Target**	**Sequence**	**%N**	**Tot.**	**19**	**Tot.**	**19**	**Reference**
1	U3, nef	AAGGCTACTTCCCTGATTGGC	35	32	33	73	75	Dave (2004)
2	U3, nef	GCTACTTCCCTGATTGGCA-GAACTA	19	22	33	62	75	Yamamoto (2006)
3	U3, nef	TTCCCTGATTGGCAGAACTACA-CAC	18	25	26	68	70	Yamamoto (2006)
4	U3, nef	GATGGTGCTTCAAGCTAGTAC	28	45	50	39	40	Capodici (2002)
5	U3, nef	AAGGAGAGAACACCAGCTTGT	0	1	1	3	5	Chang (2005)
6	U3, nef	AAGGCCCGA-GAGCTGCATCCGG	1	0	0	0	0	Suzuki (2005)
7	U3, nef	AAGACTGCTGACATCGAGCTT	1	10	10	30	30	Suzuki (2005)
8	U3	AAGGGACTTTCCGCTGGGGAC	24	28	28	87	87	Suzuki (2005)
9	U3	AACTGGGGAGTGGCGAGCCCT	0	0	0	0	0	Suzuki (2005)
10	R, TAR	GTCTCTCTGGTTAGACCAGAT	16	24	24	73	73	Yoshinari (2004)
11	R, TAR	TTAGACCAGATCTGAGCCTGG	16	24	25	81	81	Yoshinari (2004)
12	R, TAR	AGACCAGATCTGAGCCTGG	16	29	29	83	83	Jacque (2002)
13	R, TAR	CTGGGAGCTCTCTGGCTAACT	10	19	19	62	62	Yoshinari (2004)

				All clades		Clade B		
**S**	**Target**	**Sequence**	**%N**	**Tot.**	**19**	**Tot.**	**19**	**Reference**
14	R, TAR	TCTGGCTAACTAGGGAACCCA	11	19	19	65	65	Yoshinari (2004)
15	R, polyA	TGCTTAAGCCTCAATAAAGCT	85	71	71	0	0	Yoshinari (2004)
16	R, U5	AAGTAGTGTGTGCCCGTCTGT	65	49	51	92	94	Chang (2005)
17	U5, PBS	TCTCTAGCAGTGGCGCCCG	92	0	0	0	0	Nishitsuji (2006)
18	PBS	GGTGGCGCCCGAACAGG-GACCTT	17	0	0	0	0	Han (2004)
19	PBS	GTGGCGCCCGAACAGGGAC	90	0	0	0	0	Das (2004)
20	PBS	TGGCGCCCGAACAGGGACT	54	0	0	0	0	Das (2004)
21	U5, Y	GCGGAGGCTAGAAGGAGAG	88	0	0	0	0	Das (2004)
22	U5, Y, gag	GGCTAGAAGGAGAGAGATG	91	0	0	0	0	Das (2004)
23	U5, gag	AAGGAGAGAGATGGGTGCGA-GAGCGTC	93	0	0	0	0	ter brake (2006)
24	U5, gag	AGATGGGTGCGAGAGCGTC	93	0	0	0	0	Westerhout (2006b)
25	gag (p17)	GGGCAAGCAGGGAGCTAGAAC	22	27	29	52	55	Capodici (2002)
26	gag (p17)	GGGCAAGCAGGGAGCTA-GAACTT	22	0	29	0	55	Han (2004)
27	gag (p24)	GAGAACCAAGGGGAAGTGACA	44	53	64	68	77	Capodici (2002)
28	gag (p24)	AATAAAATAGTAAGAATGTA	77	66	67	61	61	ter brake (2006)
29	gag (p24)	AATAGTAAGAATGTATAGCCC	76	63	63	57	57	Chang (2005)
30	gag (p24)	AAGCATTGGGACCAGGAGCGA	0	0	2	0	4	Hu (2002)
31	gag (p24)	TAGAAGAAATGATGACAGCATG	82	76	77	76	76	ter brake (2006)
32	gag (p24)	AAGAAATGATGACAGCATGTC	62	62	77	73	76	Chang (2005)
33	gag (p7)	AAGATTGTACTGAGAGACAGG	9	20	21	43	46	Novina (2002)
34	gag (p7)	AAGATTGTACTGAGAGACAGGCT	10	19	21	40	46	Suzuki (2005)
35	gag (p7)	GATTGTACTGAGAGACAGG	9	22	22	48	48	Song (2003)
36	gag (p7)	GATTGTACTGAGAGACAGG	9	22	22	48	48	Lee (2005)
37	gag (p7)	GTACTGAGAGACAGGCTAA	9	28	28	52	52	Pusch (2003)
38	gag(p7,p1), pol	CAGGCTAATTTTTTAGGGAA	71	74	80	71	74	ter brake (2006)
39	gag (p6), pol	GAGGAGAGCTTCAGGTTTGGG	4	9	10	21	21	Lin (2004)
40	pol (prot)	AGCTCTATTAGATACAGGA	45	42	42	66	66	Paul (2003)
41	pol (prot)	GCTCTATTAGATACAGGAGCA	47	43	44	67	67	Scherer (2004)

				All clades		Clade B		
**S**	**Target**	**Sequence**	**%N**	**Tot.**	**19**	**Tot.**	**19**	**Reference**
42	pol (prot)	ACAGGAGCAGATGATACAGT	60	83	83	84	84	ter brake (2006)
43	pol (prot)	ATGGAAACCAAAAATGATAGG	82	76	76	80	80	ter brake (2006)
44	pol (RT)	CCTATTGAGACTGTACCAG	8	6	6	6	6	Paul (2003)
45	pol (RT)	AAGCCAGGAATGGATGGCCCA	64	61	62	82	82	Chang (2005)
46	pol (RT)	GACAGTACTAAATGGAGAATT	0	0	27	0	70	Sabariegos (2005)
47	pol (RT)	TGAGACACCAGGGATTAGA	23	23	23	52	52	Surabhi (2002)
48	pol (RT)	GAGACACCAGGGATTAGAT	23	24	24	53	53	Surabhi (2002)
49	pol (RT)	TCAATACATGGATGATTTGTA	27	25	25	64	65	Huelsmann (2005)
50	pol (p15)	AAGGAATTGGAGGAAATGAAC	47	50	52	68	70	Chang (2005)
51	pol (p15)	ATTGGAGGAAATGAACAAGT	48	76	76	79	79	ter brake (2006)
52	pol (int)	GGAGAGCAATGGCTAGTGA	59	53	53	59	59	Nishitsuji (2006)
53	pol (int)	AATTAGCAGGAAGATGGCCAG	67	71	73	68	69	Chang (2005)
54	pol (int)	TTAGCAGGAAGATGGCCAGT	67	78	78	78	78	ter brake (2006)
55	pol (int)	ATTCCCTACAATCCCCAAAG	51	72	72	82	82	ter brake (2006)
56	pol (int), cPPT	CACAATTTTAAAA-GAAAAGGGGGGATTGGGGGG	91	71	86	64	87	ter brake (2006)
57	pol (int)	TACAGTGCAGGGGAAAGAATA	84	79	81	82	86	ter brake (2006)
58	pol (int)	CAGTGCAGGGGAAAGAATA	84	79	79	82	82	Westerhout (2006b)
59	pol (int), CTS	AAAATTCAAAATTTTCGGGT	77	81	81	82	82	ter brake (2006)
60	pol (int), CTS	TTCAAAATTTTCGGGTTTATT	81	84	84	86	86	ter brake (2006)
61	pol (int), CTS	AATTTTCGGGTTTATTACAG	79	84	84	83	83	ter brake (2006)
62	pol (int)	CTCTGGAAAGGTGAAGGGG-CAGTAGTAAT	84	68	84	69	84	ter brake (2006)
63	pol (int)	AAGGTGAAGGGGCAGTAGTAA	70	74	84	79	91	Chang (2005)
64	pol (int)	AAGGGGCAGTAGTAATAGAAG	0	0	0	0	0	Hu (2002)
65	pol (int), vif	TATGGAAAACAGATGGCAGGTG	91	88	95	93	95	ter brake (2006)
66	vif	GGAAAGCTAAGGACTGGTT	0	0	0	0	0	Jacque (2002)
67	vif	GGAAAGCTAAGGACTGGTT	0	0	0	0	0	Joshi (2005)
68	vif	GTTCAGAAGTACACATCCC	61	58	58	73	73	Lee (2005)
69	vif	AGCACACAAGTAGACCCTGA	24	11	32	20	36	Jacque (2002)
70	vif	AGCACACAAGTAGACCCTG	24	32	32	36	36	Jacque (2002)
71	vif	AGCACACAAGTAGACCCTG	24	32	32	36	36	Nishitsuji (2004)

				All clades		Clade B		
**S**	**Target**	**Sequence**	**%N**	**Tot.**	**19**	**Tot.**	**19**	**Reference**
72	vif	CTTGGCACTAGCAGCATTA	6	17	17	33	33	Jacque (2002)
73	tat	CTAGAGCCCTGGAAGCATC	8	44	44	69	69	Surabhi (2002)
74	tat	AAGCATCCAGGAAGTCAGCCT	10	41	44	66	72	Dave (2004)
75	tat	AACTGCTTGTACCAATTGCTA	2	2	2	5	5	Coburn (2002)
76	tat	CTGCTTGTACCAATTGCTATT	5	12	12	19	19	Boden (2003a,b)
77	tat	CTGCTTGTACCAATTGCTA	3	12	12	9	9	Boden (2004b)
78	tat	CTGCTTGTACCAATTGCTATT	5	12	12	19	19	Huelsmann (2005)
79	tat	AAGTGTTGCTTTCATTGCCAAG	5	25	26	37	38	Lee (2003)
80	tat, rev	GCCTTAGGCATCTCCTATG	1	9	9	6	6	Das (2004)
81	tat, rev	TCCTATGGCAGGAA-GAAGCGGAG	82	68	80	72	77	ter brake (2006)
82	tat, rev	CCTATGGCAGGAAGAAGCG	57	80	80	77	77	Das (2004)
83	tat, rev	TATGGCAGGAAGAAGCGGA	80	82	82	78	78	Surabhi (2002)
84	tat, rev	GAAGCGGAGACAGCGACGA	46	57	57	66	66	Surabhi (2002)
85	tat, rev	GCGGAGACAGCGACGAAGAGC	7	21	41	35	59	Lee (2002)
86	tat, rev	GCGGAGACAGCGACGAAGAGC	7	21	41	35	59	Li (2003)
87	tat, rev	GCGGAGACAGCGACGAAGAGC	7	21	41	35	59	Scherer (2004)
88	tat, rev	GCGGAGACAGCGACGAAGAGC	7	21	41	35	59	Joshi (2005)
89	tat, rev	GCGGAGACAGCGACGAAGAGC	7	21	41	35	59	Li (2005)
90	tat, rev	GACGAAGAGCTCATCAGAACA	0	0	0	0	0	Boden (2004a)
91	vpu	AAGAGCAGAAGACAGTGGCAA	54	56	56	72	72	Chang (2005)
92	vpu, env	AAGACAGTGGCAATGAGAGTG	52	0	0	0	0	Chang (2005)
93	env	AAGAGGGGACCAGGGAGAG-CAC	0	0	0	0	0	Park (2003)
94	env	AAGTTCTACTGTAATTCAACAC	0	0	0	0	0	Park (2003)
95	env	CAACACAACTGTTTAATAGTA	4	15	16	27	28	Scherer (2004)
96	env	CACAATCACACTCCCATGCAG	1	7	7	12	12	Scherer (2004)
97	env	AAGACACTCCCATGCAGAATAC	0	0	0	0	0	Park (2003)
98	env	AAGATGTGGCAGGAAGTAGGAC	0	0	0	0	0	Park (2003)
99	env	GGAGGAGGCGATATGAGGGAC	0	0	0	0	0	Scherer (2004)
100	env	CAGCAGGAAGCACTATGGGCGC	76	79	80	88	89	ter brake (2006)
101	env	TGTCTGATATAGTGCAGCAGC	0	0	0	0	0	Scherer (2004)

				All clades		Clade B		
**S**	**Target**	**Sequence**	**%N**	**Tot.**	**19**	**Tot.**	**19**	**Reference**
102	env	TCTGTTGCAACTCACAGTCTG	24	45	45	65	66	Scherer (2004)
103	env	AACTCACAGTCTGGGGCATCA	12	42	64	74	82	Dave (2004)
104	tat, rev, env	AAGAAGAAGGTGGAGAGAGAG	7	30	30	52	53	Dave (2004)
105	rev, env	AACGGATCCTTGGCACTTATC	0	0	0	0	0	Coburn (2002)
106	rev, env	GGCACTTATCTGGGACGAT	0	0	0	0	0	Lee (2005)
107	rev, env	GCCTGTGCCTCTTCAGCTACC	41	55	55	65	65	Lee (2002)
108	rev, env	GCCTGTGCCTCTTCAGCTACC	41	55	55	65	65	Banerjea (2003)
109	rev, env	GCCTGTGCCTCTTCAGCTACC	41	55	55	65	65	Li (2003)
110	rev, env	GCCTGTGCCTCTTCAGCTACC	41	55	55	65	65	Scherer (2004)
111	rev, env	GCCTGTGCCTCTTCAGCTACC	41	55	55	65	65	Unwalla (2004)
112	rev, env	GCCTGTGCCTCTTCAGCTACC	41	55	55	65	65	Joshi (2005)
113	rev, env	GCCTGTGCCTCTTCAGCTACC	41	55	55	65	65	Unwalla (2006)
114	rev, env	AACGAGGATTGTGGAACTTCT	9	18	18	28	28	Dave (2004)
115	env	AAGAATAAGACAGGGCTTGGA	10	26	26	44	44	Dave (2004)
116	env	AATAAGACAGGGCTTGGAAAG	11	26	27	44	45	Dave (2004)
117	nef	AAGTAGTGTGATTGGATGGCC	0	1	1	1	1	Chang (2005)
118	nef	AAGGGAAAGAATGAGACGAGC	6	19	27	29	41	Dave (2004)
119	nef	CAUGGAGCAAUCACAAGUATT	0	0	0	0	0	Sabariegos (2005)
120	nef	GTGCCTGGCTAGAAGCACA	12	51	51	68	68	Jacque (2002)
121	nef	GTGCCTGGCTAGAAGCACA	12	51	51	68	68	Das (2004)
122	nef	GTGCCTGGCTAGAAGCACA	12	51	51	68	68	Westerhout (2005)
123	nef	GTGCCTGGCTAGAAGCACA	12	51	51	68	68	Westerhout (2006a,b)
124	nef	TTCCAGTCACACCTCAGGTAC	2	2	3	4	4	Scherer (2004)
125	nef, U3	CTTTTTAAAAGAAAAGGGGG-GACTGGA	91	75	76	72	73	ter brake (2006)
126	nef, U3	AAAAGAAAAGGGGGGACTGGA	91	79	80	77	77	Dave (2004)
127	nef, U3	GGGGGGACTGGAAGGGCTA	14	53	53	73	73	Das (2004)
128	nef, U3	GGGACTGGAAGGGCTAATT	11	33	33	47	47	Westerhout (2006b)
129	nef, U3	GACTGGAAGGGCTAATTCA	3	10	10	16	16	Nishitsuji (2006)
130	Ltr	CCCTCAGATGCTGCATATAAG	75	41	41	52	52	Naito (2007)
131	Ltr	CCTCAGATGCTGCATATAAGC	75	41	41	52	52	Naito (2007)

				All clades		Clade B		
**S**	**Target**	**Sequence**	**%N**	**Tot.**	**19**	**Tot.**	**19**	**Reference**
132	Ltr	CTCAGATGCTGCATATAAGCA	75	41	41	51	52	Naito (2007)
133	Ltr	TCAGATGCTGCATATAAGCAG	75	41	41	51	52	Naito (2007)
134	Ltr	CAGATGCTGCATATAAGCAGC	76	41	41	51	51	Naito (2007)
135	Ltr	GGGAACCCACTGCTTAAGCCT	78	64	64	97	97	Naito (2007)
136	Ltr	GGAACCCACTGCTTAAGCCTC	81	67	67	97	97	Naito (2007)
137	Ltr	GAACCCACTGCTTAAGCCTCA	85	69	69	98	98	Naito (2007)
138	Ltr	AACCCACTGCTTAAGCCTCAA	85	69	69	100	100	Naito (2007)
139	Ltr	ACCCACTGCTTAAGCCTCAAT	85	69	69	100	100	Naito (2007)
140	Ltr	CCCACTGCTTAAGCCTCAATA	85	70	70	100	100	Naito (2007)
141	Ltr	CCACTGCTTAAGCCTCAATAA	85	71	71	100	100	Naito (2007)
142	Ltr	CACTGCTTAAGCCTCAATAAA	85	71	71	100	100	Naito (2007)
143	Ltr	ACTGCTTAAGCCTCAATAAAG	85	71	71	100	100	Naito (2007)
144	Ltr	CTGCTTAAGCCTCAATAAAGC	85	71	71	100	100	Naito (2007)
145	Ltr	TGCTTAAGCCTCAATAAAGCT	85	71	71	100	100	Naito (2007)
146	Ltr	GCTTAAGCCTCAATAAAGCTT	86	71	71	100	100	Naito (2007)
147	Ltr	CTTAAGCCTCAATAAAGCTTG	86	71	71	100	100	Naito (2007)
148	Ltr	TTAAGCCTCAATAAAGCTTGC	87	71	71	100	100	Naito (2007)
149	Ltr	TAAGCCTCAATAAAGCTTGCC	87	71	71	100	100	Naito (2007)
150	Ltr	AAGCCTCAATAAAGCTTGCCT	94	90	93	100	100	Naito (2007)
151	Ltr	AGCCTCAATAAAGCTTGCCTT	94	90	91	100	100	Naito (2007)
152	Ltr	GCCTCAATAAAGCTTGCCTTG	95	91	92	100	100	Naito (2007)
153	Ltr	CCTCAATAAAGCTTGCCTTGA	94	92	92	100	100	Naito (2007)
154	Ltr	CTCAATAAAGCTTGCCTTGAG	95	93	93	100	100	Naito (2007)
155	Ltr	TCAATAAAGCTTGCCTTGAGT	88	93	93	100	100	Naito (2007)
156	Ltr	CAATAAAGCTTGCCTTGAGTG	88	93	93	100	100	Naito (2007)
157	Ltr	AATAAAGCTTGCCTTGAGTGC	86	91	93	100	100	Naito (2007)
158	Ltr	ATAAAGCTTGCCTTGAGTGCT	80	89	93	99	100	Naito (2007)
159	Ltr	AGTAGTGTGTGCCCGTCTGTT	70	50	52	92	92	Naito (2007)
160	Ltr	TGACTCTGGTAACTAGAGATC	72	52	53	90	90	Naito (2007)
161	Ltr	GACTCTGGTAACTAGAGATCC	81	89	90	90	90	Naito (2007)
162	Ltr	ACTCTGGTAACTAGAGATCCC	82	91	91	90	90	Naito (2007)
163	Ltr	CTCTGGTAACTAGAGATCCCT	87	91	91	90	90	Naito (2007)
164	Ltr	TCTGGTAACTAGAGATCCCTC	87	91	91	90	90	Naito (2007)

				All clades		Clade B		
**S**	**Target**	**Sequence**	**%N**	**Tot.**	**19**	**Tot.**	**19**	**Reference**
165	Ltr	CTGGTAACTAGAGATCCCTCA	88	91	91	90	90	Naito (2007)
166	Ltr	TGGTAACTAGAGATCCCTCAG	88	91	91	90	90	Naito (2007)
167	Ltr	GGTAACTAGAGATCCCTCAGA	88	90	91	89	90	Naito (2007)
168	Ltr	GTAACTAGAGATCCCTCAGAC	88	89	91	86	90	Naito (2007)
169	Ltr	TAACTAGAGATCCCTCAGACC	84	84	91	83	91	Naito (2007)
170	Ltr	AAAATCTCTAGCAGTGGCGCC	90	0	0	0	0	Naito (2007)
171	Ltr	AAATCTCTAGCAGTGGCGCCC	93	0	0	0	0	Naito (2007)
172	Ltr	AATCTCTAGCAGTGGCGCCCG	92	0	0	0	0	Naito (2007)
173	Ltr	ATCTCTAGCAGTGGCGCCCGA	92	0	0	0	0	Naito (2007)
174	Ltr	TCTCTAGCAGTGGCGCCCGAA	93	0	0	0	0	Naito (2007)
175	Ltr	CTCTAGCAGTGGCGCCCGAAC	93	0	0	0	0	Naito (2007)
176	Ltr	TCTAGCAGTGGCGCCCGAACA	92	0	0	0	0	Naito (2007)
177	Ltr	CTAGCAGTGGCGCCCGAACAG	92	0	0	0	0	Naito (2007)
178	Ltr	TAGCAGTGGCGCCCGAACAGG	94	0	0	0	0	Naito (2007)
179	Ltr	AGCAGTGGCGCCCGAACAGGG	93	0	0	0	0	Naito (2007)
180	Ltr	GCAGTGGCGCCCGAACAGGGA	90	0	0	0	0	Naito (2007)
181	Ltr	CAGTGGCGCCCGAACAGGGAC	90	0	0	0	0	Naito (2007)
182	Ltr	TCTCTCGACGCAGGACTCGGC	85	0	0	0	0	Naito (2007)
183	Ltr	CTCTCGACGCAGGACTCGGCT	88	0	0	0	0	Naito (2007)
184	Ltr	TCTCGACGCAGGACTCGGCTT	89	0	0	0	0	Naito (2007)
185	Ltr	CTCGACGCAGGACTCGGCTTG	89	0	0	0	0	Naito (2007)
186	Ltr	TCGACGCAGGACTCGGCTTGC	88	0	0	0	0	Naito (2007)
187	Ltr	CGACGCAGGACTCGGCTTGCT	89	0	0	0	0	Naito (2007)
188	Ltr	GACGCAGGACTCGGCTTGCTG	90	0	0	0	0	Naito (2007)
189	Ltr	ACGCAGGACTCGGCTTGCTGA	88	0	0	0	0	Naito (2007)
190	Ltr	TTTGACTAGCGGAGGCTAGAA	85	0	0	0	0	Naito (2007)
191	Ltr	TTGACTAGCGGAGGCTAGAAG	87	0	0	0	0	Naito (2007)
192	Ltr	TGACTAGCGGAGGCTAGAAGG	88	0	0	0	0	Naito (2007)
193	Ltr	GACTAGCGGAGGCTAGAAGGA	88	0	0	0	0	Naito (2007)
194	Ltr	ACTAGCGGAGGCTAGAAGGAG	88	0	0	0	0	Naito (2007)
195	Ltr	CTAGCGGAGGCTAGAAGGAGA	88	0	0	0	0	Naito (2007)
196	Ltr	TAGCGGAGGCTAGAAGGAGAG	88	0	0	0	0	Naito (2007)
197	Ltr	AGCGGAGGCTAGAAGGAGAGA	88	0	0	0	0	Naito (2007)

				All clades		Clade B		
**S**	**Target**	**Sequence**	**%N**	**Tot.**	**19**	**Tot.**	**19**	**Reference**
198	Ltr	GCGGAGGCTAGAAGGAGAGAG	88	0	0	0	0	Naito (2007)
199	Ltr	CGGAGGCTAGAAGGAGAGAGA	89	0	0	0	0	Naito (2007)
200	Ltr	GGAGGCTAGAAGGAGAGAGAT	88	0	0	0	0	Naito (2007)
201	Ltr	GAGGCTAGAAGGAGAGAGATG	91	0	0	0	0	Naito (2007)
202	Ltr	AGGCTAGAAGGAGAGAGATGG	91	0	0	0	0	Naito (2007)
203	Ltr	GGCTAGAAGGAGAGAGATGGG	91	0	0	0	0	Naito (2007)
204	Ltr	GCTAGAAGGAGAGAGATGGGT	91	0	0	0	0	Naito (2007)
205	Ltr	CTAGAAGGAGAGAGATGGGTG	90	0	0	0	0	Naito (2007)
206	Ltr	TAGAAGGAGAGAGATGGGTGC	90	0	0	0	0	Naito (2007)
207	Ltr	AGAAGGAGAGAGATGGGTGCG	90	0	0	0	0	Naito (2007)
208	Ltr	GAAGGAGAGAGATGGGTGCGA	90	0	0	0	0	Naito (2007)
209	Ltr	AAGGAGAGAGATGGGTGCGAG	92	0	0	0	0	Naito (2007)
210	Ltr	AGGAGAGAGATGGGTGCGAGA	92	0	0	0	0	Naito (2007)
211	Ltr	GGAGAGAGATGGGTGCGAGAG	92	0	0	0	0	Naito (2007)
212	Ltr	GAGAGAGATGGGTGCGAGAGC	93	0	0	0	0	Naito (2007)
213	Ltr	AGAGAGATGGGTGCGAGAGCG	93	0	0	0	0	Naito (2007)
214	Ltr	GAGAGATGGGTGCGAGAGCGT	93	0	0	0	0	Naito (2007)
215	Ltr	AGAGATGGGTGCGAGAGCGTC	93	0	0	0	0	Naito (2007)
216	Ltr	GAGATGGGTGCGAGAGCGTCA	90	0	0	0	0	Naito (2007)
217	Gag	TTAAATAAAATAGTAAGAATG	72	63	63	59	59	Naito (2007)
218	Gag	TAAATAAAATAGTAAGAATGT	77	65	65	60	60	Naito (2007)
219	Gag	AAATAAAATAGTAAGAATGTA	77	65	65	60	60	Naito (2007)
220	Gag	AATAAAATAGTAAGAATGTAT	78	65	67	60	61	Naito (2007)
221	Gag	ATAAAATAGTAAGAATGTATA	78	65	67	60	61	Naito (2007)
222	Gag	TAAAATAGTAAGAATGTATAG	78	65	65	60	60	Naito (2007)
223	Gag	AAAATAGTAAGAATGTATAGC	76	63	65	57	60	Naito (2007)
224	Gag	AAATAGTAAGAATGTATAGCC	76	63	65	57	60	Naito (2007)
225	Gag	AATAGTAAGAATGTATAGCCC	76	63	63	57	57	Naito (2007)
226	Gag	ATAGTAAGAATGTATAGCCCT	76	62	65	56	58	Naito (2007)
227	Gag	TAGAAGAAATGATGACAGCAT	82	76	77	76	76	Naito (2007)
228	Gag	AGAAGAAATGATGACAGCATG	82	76	76	76	76	Naito (2007)
229	Gag	GACAGGCTAATTTTTTAGGGA	71	58	60	71	74	Naito (2007)
230	Gag	CAGGCTAATTTTTTAGGGAAA	70	58	80	42	74	Naito (2007)

				All clades		Clade B		
**S**	**Target**	**Sequence**	**%N**	**Tot.**	**19**	**Tot.**	**19**	**Reference**
231	Gag	TCCCTCAAATCACTCTTTGGC	80	67	67	51	51	Naito (2007)
232	Gag	CCCTCAAATCACTCTTTGGCA	82	69	69	52	52	Naito (2007)
233	Pol	ACAGGAGCAGATGATACAGTA	81	80	83	81	84	Naito (2007)
234	Pol	CAGGAGCAGATGATACAGTAT	78	76	83	78	84	Naito (2007)
235	Pol	AGGAGCAGATGATACAGTATT	78	76	80	78	81	Naito (2007)
236	Pol	GGAGCAGATGATACAGTATTA	79	79	80	86	87	Naito (2007)
237	Pol	GAGCAGATGATACAGTATTAG	79	78	80	86	87	Naito (2007)
238	Pol	AGCAGATGATACAGTATTAGA	77	78	79	85	86	Naito (2007)
239	Pol	GCAGATGATACAGTATTAGAA	80	79	80	86	87	Naito (2007)
240	Pol	CAGATGATACAGTATTAGAAG	78	77	80	86	87	Naito (2007)
241	Pol	AGATGATACAGTATTAGAAGA	78	77	79	86	87	Naito (2007)
242	Pol	ATGGAAACCAAAAATGATAGG	82	76	76	80	80	Naito (2007)
243	Pol	CCTACACCTGTCAACATAATT	72	66	67	65	65	Naito (2007)
244	Pol	CTACACCTGTCAACATAATTG	72	67	67	65	65	Naito (2007)
245	Pol	TACACCTGTCAACATAATTGG	72	67	67	65	65	Naito (2007)
246	Pol	ACACCTGTCAACATAATTGGA	73	66	70	66	68	Naito (2007)
247	Pol	TTAAACAATGGCCATTGACAG	70	64	64	80	80	Naito (2007)
248	Pol	AAACAATGGCCATTGACAGAA	73	66	68	81	84	Naito (2007)
249	Pol	AACAATGGCCATTGACAGAAG	73	66	68	81	84	Naito (2007)
250	Pol	ACAATGGCCATTGACAGAAGA	74	66	66	81	81	Naito (2007)
251	Pol	AATTGGGCCTGAAAATCCATA	75	76	76	81	81	Naito (2007)
252	Pol	CTGGACTGTCAATGATATACA	70	59	59	15	15	Naito (2007)
253	Pol	ATTGGAGGAAATGAACAAGTA	77	74	76	78	79	Naito (2007)
254	Pol	TTGGAGGAAATGAACAAGTAG	77	74	76	77	79	Naito (2007)
255	Pol	TGGAGGAAATGAACAAGTAGA	77	74	74	77	78	Naito (2007)
256	Pol	GGAGGAAATGAACAAGTAGAT	71	71	76	77	79	Naito (2007)
257	Pol	GAGGAAATGAACAAGTAGATA	71	70	76	76	79	Naito (2007)
258	Pol	AGGAAATGAACAAGTAGATAA	71	70	71	76	77	Naito (2007)
259	Pol	GGAAATGAACAAGTAGATAAA	71	72	73	76	78	Naito (2007)
260	Pol	AATGAACAAGTAGATAAATTA	72	72	73	76	77	Naito (2007)
261	Pol	ATGAACAAGTAGATAAATTAG	72	73	73	76	77	Naito (2007)
262	Pol	TGAACAAGTAGATAAATTAGT	72	72	73	76	76	Naito (2007)
263	Pol	ACAGCAGTACAAATGGCAGTA	75	79	82	85	87	Naito (2007)

				All clades		Clade B		
**S**	**Target**	**Sequence**	**%N**	**Tot.**	**19**	**Tot.**	**19**	**Reference**
264	Pol	CAGCAGTACAAATGGCAGTAT	74	78	82	84	87	Naito (2007)
265	Pol	AGCAGTACAAATGGCAGTATT	74	78	79	84	85	Naito (2007)
266	Pol	GCAGTACAAATGGCAGTATTC	72	73	78	74	84	Naito (2007)
267	Pol	CAGTACAAATGGCAGTATTCA	72	72	78	73	84	Naito (2007)
268	Pol	AGTACAAATGGCAGTATTCAT	72	73	73	74	74	Naito (2007)
269	Pol	CACAATTTTAAAAGAAAAGGG	90	86	86	87	87	Naito (2007)
270	Pol	ACAATTTTAAAAGAAAAGGGG	90	85	86	87	87	Naito (2007)
271	Pol	CAATTTTAAAAGAAAAGGGGG	90	86	86	87	87	Naito (2007)
272	Pol	AATTTTAAAAGAAAAGGGGGG	91	88	89	87	87	Naito (2007)
273	Pol	ATTTTAAAAGAAAAGGGGGGA	91	88	88	87	87	Naito (2007)
274	Pol	TTTTAAAAGAAAAGGGGGGAT	91	88	88	87	87	Naito (2007)
275	Pol	TTTAAAAGAAAAGGGGGGATT	91	88	88	87	87	Naito (2007)
276	Pol	TTAAAAGAAAAGGGGGGATTG	91	88	88	87	87	Naito (2007)
277	Pol	TAAAAGAAAAGGGGGGATTGG	91	89	89	88	88	Naito (2007)
278	Pol	AAAAGAAAAGGGGGGATTGGG	90	88	89	87	88	Naito (2007)
279	Pol	AAAGAAAAGGGGGGATTGGGG	89	88	89	87	88	Naito (2007)
280	Pol	AAGAAAAGGGGGGATTGGGGG	87	85	88	83	88	Naito (2007)
281	Pol	AGAAAAGGGGGGATTGGGGGG	78	77	91	69	94	Naito (2007)
282	Pol	GAAAAGGGGGGATTGGGGGGT	78	77	88	69	90	Naito (2007)
283	Pol	AAAAGGGGGGATTGGGGGGTA	78	77	78	70	70	Naito (2007)
284	Pol	AAAGGGGGGATTGGGGGGTAC	77	75	78	67	70	Naito (2007)
285	Pol	AAGGGGGGATTGGGGGGTACA	77	75	78	66	70	Naito (2007)
286	Pol	AGGGGGGATTGGGGGGTACAG	80	76	78	66	67	Naito (2007)
287	Pol	GGGGGGATTGGGGGGTACAGT	80	76	78	65	67	Naito (2007)
288	Pol	GGGGGATTGGGGGGTACAGTG	80	76	76	65	66	Naito (2007)
289	Pol	GGGGATTGGGGGGTACAGTGC	80	76	76	65	66	Naito (2007)
290	Pol	GGGATTGGGGGGTACAGTGCA	79	75	76	65	65	Naito (2007)
291	Pol	GGATTGGGGGGTACAGTGCAG	79	75	76	65	65	Naito (2007)
292	Pol	GATTGGGGGGTACAGTGCAGG	79	75	75	65	65	Naito (2007)
293	Pol	ATTGGGGGGTACAGTGCAGGG	76	72	76	63	65	Naito (2007)
294	Pol	TTGGGGGGTACAGTGCAGGGG	76	72	76	63	65	Naito (2007)
295	Pol	TGGGGGGTACAGTGCAGGGGA	76	72	72	62	63	Naito (2007)
296	Pol	GGGGGGTACAGTGCAGGGGAA	74	70	72	62	63	Naito (2007)

				All clades		Clade B		
**S**	**Target**	**Sequence**	**%N**	**Tot.**	**19**	**Tot.**	**19**	**Reference**
297	Pol	GGGGGTACAGTGCAGGGGAAA	73	70	72	62	62	Naito (2007)
298	Pol	GGGGTACAGTGCAGGGGAAAG	73	70	70	62	62	Naito (2007)
299	Pol	GGGTACAGTGCAGGGGAAAGA	72	69	70	62	63	Naito (2007)
300	Pol	GGTACAGTGCAGGGGAAAGAA	73	69	71	63	63	Naito (2007)
301	Pol	GTACAGTGCAGGGGAAAGAAT	75	71	71	67	67	Naito (2007)
302	Pol	TACAGTGCAGGGGAAAGAATA	84	79	81	82	86	Naito (2007)
303	Pol	ACAGTGCAGGGGAAAGAATAA	72	61	81	32	86	Naito (2007)
304	Pol	CAGTGCAGGGGAAAGAATAAT	72	61	79	32	82	Naito (2007)
305	Pol	AGTGCAGGGGAAAGAATAATA	74	65	65	34	34	Naito (2007)
306	Pol	GTGCAGGGGAAAGAATAATAG	74	65	65	34	34	Naito (2007)
307	Pol	TGCAGGGGAAAGAATAATAGA	74	66	66	34	34	Naito (2007)
308	Pol	AAAAATTCAAAATTTTCGGGT	77	80	80	82	82	Naito (2007)
309	Pol	AAAATTCAAAATTTTCGGGTT	77	79	81	81	82	Naito (2007)
310	Pol	AAATTCAAAATTTTCGGGTTT	79	81	83	81	82	Naito (2007)
311	Pol	AATTCAAAATTTTCGGGTTTA	79	81	81	82	82	Naito (2007)
312	Pol	ATTCAAAATTTTCGGGTTTAT	79	82	82	83	83	Naito (2007)
313	Pol	TTCAAAATTTTCGGGTTTATT	81	84	84	86	86	Naito (2007)
314	Pol	TCAAAATTTTCGGGTTTATTA	77	81	84	80	86	Naito (2007)
315	Pol	CAAAATTTTCGGGTTTATTAC	78	82	86	82	88	Naito (2007)
316	Pol	AAAATTTTCGGGTTTATTACA	78	82	82	82	83	Naito (2007)
317	Pol	AAATTTTCGGGTTTATTACAG	79	83	84	83	84	Naito (2007)
318	Pol	CTCTGGAAAGGTGAAGGGGCA	82	82	84	84	84	Naito (2007)
319	Pol	TCTGGAAAGGTGAAGGGGCAG	82	81	84	83	85	Naito (2007)
320	Pol	CTGGAAAGGTGAAGGGGCAGT	82	81	82	83	84	Naito (2007)
321	Pol	TGGAAAGGTGAAGGGGCAGTA	84	85	88	93	95	Naito (2007)
322	Pol	GGAAAGGTGAAGGGGCAGTAG	84	84	88	91	95	Naito (2007)
323	Pol	GAAAGGTGAAGGGGCAGTAGT	83	84	85	91	93	Naito (2007)
324	Pol	AAAGGTGAAGGGGCAGTAGTA	72	76	84	80	91	Naito (2007)
325	Pol	AGGTGAAGGGGCAGTAGTAAT	70	74	76	79	80	Naito (2007)
326	Vif	TATGGAAAACAGATGGCAGGT	91	0	0	0	0	Naito (2007)
327	Vif	ATGGAAAACAGATGGCAGGTG	90	89	95	93	95	Naito (2007)
328	Vif	TCCTATGGCAGGAAGAAGCGG	82	3	3	2	2	Naito (2007)
329	Vif	CCTATGGCAGGAAGAAGCGGA	80	2	3	2	2	Naito (2007)

			All clades		Clade B		
**S**	**Target**	**Sequence**	**%N**	**Tot.**	**19**	**Tot.**	**19**	**Reference**
330	Vif	ATGGCAGGAAGAAGCGGAGAC	70	2	3	3	3	Naito (2007)
331	Env	ACAATTGGAGAAGTGAATTAT	71	73	74	77	77	Naito (2007)
332	Env	CAATTGGAGAAGTGAATTATA	71	73	74	77	77	Naito (2007)
333	Env	AGCAGCAGGAAGCACTATGGG	71	75	75	84	84	Naito (2007)
334	Env	GCAGCAGGAAGCACTATGGGC	72	76	76	84	84	Naito (2007)
335	Env	CAGCAGGAAGCACTATGGGCG	75	79	80	88	89	Naito (2007)
336	Env	AGCAGGAAGCACTATGGGCGC	76	81	82	90	91	Naito (2007)
337	Env	ACGCTGACGGTACAGGCCAGA	72	75	78	76	79	Naito (2007)
338	Env	CGCTGACGGTACAGGCCAGAC	73	78	82	82	86	Naito (2007)
339	Nef	CTTTTTAAAAGAAAAGGGGGG	86	76	76	73	73	Naito (2007)
340	Nef	TTTTTAAAAGAAAAGGGGGGA	90	78	78	74	75	Naito (2007)
341	Nef	TTTTAAAAGAAAAGGGGGGAC	90	78	79	75	76	Naito (2007)
342	Nef	TTTAAAAGAAAAGGGGGGACT	90	79	79	76	76	Naito (2007)
343	Nef	TTAAAAGAAAAGGGGGGACTG	90	79	79	76	76	Naito (2007)
344	Nef	TAAAAGAAAAGGGGGGACTGG	91	79	80	77	77	Naito (2007)
345	Nef	AAAAGAAAAGGGGGGACTGGA	91	79	80	77	77	Naito (2007)

**Averages w/o Naito *et. al.*(103 sequences): ***	**34**	**42**	**45**	**55**	**59**
**# of shRNA > 70 % conserved**	**188**	**145**	**154**	**149**	**163**
**Averages all (270 sequences): ****	**63**	**62**	**64**	**68**	**71**
**# of shRNA > 70 % conserved**	**20**	**22**	**25**	**32**	**39**

** Twenty six of the 129 sequences published prior to Naito et. al. were excluded from this total as their conservations were unable to be estimated by one of the methods used, or they were 0 % conserved.*

*** Seventy five of the 345 sequences were excluded from this total as their conservations were unable to be estimated by one of the methods used, or they were 0 % conserved.*

Bibliography

1. Banerjea A, Li MJ, Bauer G, Remling L, Lee NS, Rossi J, Akkina R: Inhibition of HIV-1 by lentiviral vector-transduced siRNAs in T lymphocytes differentiated in SCID-hu mice and CD34+ progenitor cell-derived macrophages. *Mol Ther* 2003, 8:62-71.

2. Boden D, Pusch O, Lee F, Tucker L, Shank PR, Ramratnam B: Promoter choice affects the potency of HIV-1 specific RNA interference. *Nucleic Acids Res* 2003a, 31:5033-5038.
3. Boden D, Pusch O, Lee F, Tucker L, Ramratnam B: Human immunodeficiency virus type 1 escape from RNA interference. *J Virol* 2003b, 77:11531-11535.
4. Boden D, Pusch O, Silbermann R, Lee F, Tucker L, Ramratnam B: Enhanced gene silencing of HIV-1 specific siRNA using microRNA designed hairpins. *Nucleic Acids Res* 2004a, 32:1154-1158.
5. Boden D, Pusch O, Lee F, Tucker L, Ramratnam B: Efficient gene transfer of HIV-1-specific short hairpin RNA into human lymphocytic cells using recombinant adeno-associated virus vectors. *Mol Ther* 2004b, 9:396-402.
6. Capodici J, Karikó K, Weissman D: Inhibition of HIV-1 infection by small interfering RNA-mediated RNA interference. *J Immunol* 2002, 169:5196-5201.
7. Chang LJ, Liu X, He J: Lentiviral siRNAs targeting multiple highly conserved RNA sequences of human immunodeficiency virus type 1. *Gene Ther* 2005, 12:1133-1144.
8. Coburn GA, Cullen BR: Potent and specific inhibition of human immunodeficiency virus type 1 replication by RNA interference. *J Virol* 2002, 76:9225-9231.
9. Das AT, Brummelkamp TR, Westerhout EM, Vink M, Madiredjo M, Bernards R, Berkhout B: Human immunodeficiency virus type 1 escapes from RNA interference-mediated inhibition. *J Virol* 2004, 78:2601-2605.
10. Dave RS, Pomerantz RJ: Antiviral effects of human immunodeficiency virus type 1-specific small interfering RNAs against targets conserved in select neurotropic viral strains. *J Virol* 2004, 78:13687-13696.
11. Han W, Wind-Rotolo M, Kirkman RL, Morrow CD: Inhibition of human immunodeficiency virus type 1 replication by siRNA targeted to the highly conserved primer binding site. *Virology* 2004, 330:221-232.
12. Hu WY, Myers CP, Kilzer JM, Pfaff SL, Bushman FD: Inhibition of retroviral pathogenesis by RNA interference. *Curr Biol* 2002, 12:1301-1311.
13. Huelsmann PM, Rauch P, Allers K, John MJ, Metzner KJ: Inhibition of drug-resistant HIV-1 by RNA interference. *Antiviral Res* 2006, 69:1-8.
14. Jacque JM, Triques K, Stevenson M: Modulation of HIV-1 replication by RNA interference. *Nature* 2002, 418:435-438.
15. Joshi PJ, North TW, Prasad VR: Aptamers directed to HIV-1 reverse transcriptase display greater efficacy over small hairpin RNAs targeted to viral RNA in blocking HIV-1 replication. *Mol Ther* 2005, 11:677-686.
16. Lee MTM, Coburn GA, McClure MO, Cullen BR: Inhibition of human immunodeficiency virus type 1 replication in primary macrophages by using Tat- or CCR5-specific small interfering RNAs expressed from a lentivirus vector. *J Virol* 2003, 77:11964-11972.
17. Lee NS, Dohjima T, Bauer G, Li H, Li MJ, Ehsani A, Salvaterra P, Rossi J: Expression of small interfering RNAs targeted against HIV-1 *rev* transcripts in human cells. *Nat Biotechnol* 2002, 20:500-505.

18. Lee SK, Dykxhoorn DM, Kumar P, Ranjbar S, Song E, Maliszewski LE, François-Bongarçon V, Goldfeld A, Swamy NM, Lieberman J, Shankar P: Lentiviral delivery of short hairpin RNAs protects CD4 T cells from multiple clades and primary isolates of HIV. *Blood* 2005, 106:818-826.
19. Li MJ, Bauer G, Michienzi A, Yee JK, Lee NS, Kim J, Li S, Castanotto D, Zaia J, Rossi JJ: Inhibition of HIV-1 infection by lentiviral vectors expressing Pol III-promoted anti-HIV RNAs. *Mol Ther* 2003, 8:196-206.
20. Li MJ, Kim J, Li S, Zaia J, Yee JK, Anderson J, Akkina R, Rossi JJ: Long-term inhibition of HIV-1 infection in primary hematopoietic cells by lentiviral vector delivery of a triple combination of anti-HIV shRNA, anti-CCR5 ribozyme, and a nucleolar-localizing TAR decoy. *Mol Ther* 2005, 12:900-909.
21. Lin SL, Ying SY: Combinational therapy for potential HIV-1 eradication and vaccination. *Int J Oncol* 2004, 24:81-88.
22. Nishitsuji H, Ikeda T, Miyoshi H, Ohashi T, Kannagi M, Masuda T: Expression of small hairpin RNA by lentivirus-based vector confers efficient and stable gene-suppression of HIV-1 on human cells including primary non-dividing cells. *Microbes Infect* 2004, 6:76-85.
23. Nishitsuji H, Kohara M, Kannagi M, Masuda T: Effective suppression of human immunodeficiency virus type 1 through a combination of short- or long-hairpin RNAs targeting essential sequences for retroviral integration. *J Virol* 2006, 80:7658-7666.
24. Novina CD, Murray MF, Dykxhoorn DM, Beresford PJ, Riess J, Lee SK, Collman RG, Lieberman J, Shankar P, Sharp PA: siRNA-directed inhibition of HIV-1 infection. *Nat Med* 2002, 8:681-686.
25. Park WS, Hayafune M, Miyano-Kurosaki N, Takaku H: Specific HIV-1 env gene silencing by small interfering RNAs in human peripheral blood mononuclear cells. *Gene Ther* 2003, 10:2046-2050.
26. Paul CP, Good PD, Li SXL, Kleihauer A, Rossi JJ, Engelke DR: Localized expression of small RNA inhibitors in human cells. *Mol Ther* 2003, 7:237-247.
27. Pusch O, Boden D, Silbermann R, Lee F, Tucker L, Ramratnam B: Nucleotide sequence homology requirements of HIV-1-specific short hairpin RNA. *Nucleic Acids Res* 2003, 31:6444-6449.
28. Sabariegos R, Giménez-Barcons M, Tàpia N, Clotet B, Martínez MA: Sequence homology required by human immunodeficiency virus type 1 to escape from short interfering RNAs. *J Virol* 2006, 80:571-577.
29. Scherer LJ, Yildiz Y, Kim J, Cagnon L, Heale B, Rossi JJ: Rapid assessment of anti-HIV siRNA efficacy using PCR-derived Pol III shRNA cassettes. *Mol Ther* 2004, 10:597-603.
30. Song E, Lee SK, Dykxhoorn DM, Novina C, Zhang D, Crawford K, Cerny J, Sharp PA, Lieberman J, Manjunath N, Shankar P: Sustained small interfering RNA-mediated human immunodeficiency virus type 1 inhibition in primary macrophages. *J Virol* 2003, 77:7174-7181.

31. Surabhi RM, Gaynor RB: RNA interference directed against viral and cellular targets inhibits human immunodeficiency virus type 1 replication. *J Virol* 2002, 76:12963-12973.

32. Suzuki K, Shijuuku T, Fukamachi T, Zaunders J, Guillemin G, Cooper D, Kelleher A: Prolonged transcriptional silencing and CpG methylation induced by siRNAs targeted to the HIV-1 promoter region. J RNAi Gene Silencing 2005, 1:66-78.

33. ter Brake O, Konstantinova P, Ceylan M, Berkhout B: Silencing of HIV-1 with RNA interference: a multiple shRNA approach. *Mol Ther* 2006, 14:883-892.

34. Unwalla HJ, Li MJ, Kim JD, Li HT, Ehsani A, Alluin J, Rossi JJ: Negative feedback inhibition of HIV-1 by TAT-inducible expression of siRNA. *Nat Biotechnol* 2004, 22:1573-1578.

35. Unwalla HJ, Li HT, Bahner I, Li MJ, Kohn D, Rossi JJ: Novel Pol II fusion promoter directs human immunodeficiency virus type 1-inducible coexpression of a short hairpin RNA and protein. *J Virol* 2006, 80:1863-1873.

36. Westerhout EM, Ooms M, Vink M, Das AT, Berkhout B: HIV-1 can escape from RNA interference by evolving an alternative structure in its RNA genome. *Nucleic Acids Res* 2005, 33:796-804.

37. Westerhout EM, Vink M, Haasnoot PCJ, Das AT, Berkhout B: A conditionally replicating HIV-based vector that stably expresses an antiviral shRNA against HIV-1 replication. *Mol Ther* 2006a, 14:268-275.

38. Westerhout EM, ter Brake O, Berkhout B: The virion-associated incoming HIV-1 RNA genome is not targeted by RNA interference. *Retrovirology* 2006b, 3:57.

39. Yamamoto T, Miyoshi H, Yamamoto N, Yamamoto N, Inoue J, Tsunetsugu-Yokota Y: Lentivirus vectors expressing short hairpin RNAs against the *U3*-overlapping region of HIV *nef* inhibit HIV replication and infectivity in primary macrophages. *Blood* 2006, 108:3305-3312.

40. Yoshinari K, Miyagishi M, Taira K: Effects on RNAi of the tight structure, sequence and position of the targeted region. *Nucleic Acids Res* 2004, 32:691-699.
