# Supplementary material for: 96 shRNAs designed for maximal coverage of HIV-1 variants
Source: Retrovirology. 2009 Jun 4;6:55. doi: 10.1186/1742-4690-6-55 (PMC2698899; doi:10.1186/1742-4690-6-55)
Supplement: Additional file 2 — An additional data file containing (i) a table of the the number of sequences in the HIV-1 partial gene fragments data set, (ii) a table summarizing the number of 19 nt. sequences analyzed, (iii) a summary table of the numbers of most highly conserved targets, (iv) a list of the 96 selected targets and surrounding sequence, (v) a table of the 22 overlapping regions containing the 96 targets, (vi) a list of the 20 and 21 bp matched targets, (vii) a list of the overlapping hairpins with adjacent start sites 1 nt. apart, (viii) a detailed list of the % conservation for each position in the 5 sequence profile for all 96 selected targets, (ix) a table summarizing the differences in the fluorescent reporters used, (x) a list of the hairpins in scored and ranked order, (xi) additional method information such as oligonucleotide sequences, and (xii) the NL4-3 gene sequences used to generate the targets and make the reporters. [file 1742-4690-6-55-S2.pdf]

The HIV sequence sets

The following table shows the number of individual sequences in each gene set from LANL, Virco and both combined. It also shows the number and percentage of LANL sequences that were clade B, as well as the average length of the sequences from each LANL gene set compared to the corresponding gene length from NL4-3. The LANL sequences are available on request.

Gene	LANL (all)	Virco	LANL (+V.)	LANL (B)	LANL % B	LANL len.	NL4-3 len.
LTR	382		382	111	29%	636	634
Gag	1610		1610	748	46%	1497	1503
Pol	1567		1567	438	28%	3007	3012
Vif	2507	105	2612	1253	48%	579	579
Vpr	2319	105	2424	998	41%	291	**288 (291) ***
Tat (x1)	3262	105	3367	1500	45%	215	215
Rev (x1)	3403	105	3508	1530	44%	76	76
Vpu	2442	105	2547	886	35%	251	**243 (246) ***
Env	3560		3560	2026	57%	2574	2565
Tat (x2)	5781	83	5864	2961	50%	91	46
Rev (x2)	5469	83	5552	2771	50%	280	275
Nef	4873	83	4956	3106	63%	622	**618 (621) ***
**Totals :**	**37175**	**774**	**37949**	**18328**	**45%**		

nb. The Virco data included a small amount of LTR sequence with the Nef data, which as used where possible, but not included in the above sequence tallies.

** the native 3 nt. stop codons (the last 3 nucleotides) were excluded from the individual gene fragment sequences used in this study as it they were not included our reporters, however, the missing 3 nt. sequences were included in overlapping gene fragments.*

The 19 mer target sets

If the complete genome for the NL4-3 strain was broken into all possible 19 mers there would be **9, 691** sequences (nb. including duplicate LTR sequences). By using individual gene sets we generated **9, 838** 19 mer sequences. This included **936** duplicate and **28** triplicate sequences from partially overlapping genes. There were also **227** sequences missing (or **2.3** % of all possible 19 mers) due to several inter-gene gaps. Notably, this included **173** sequences between the 5' LTR and Gag (617 - 789; NL4-3 co-ordinates) which covered the highly structured packaging signal **psi** (**Ψ**). However, this region was covered by Naito *et. al.* (2007), using full-length genomes to estimate conservations. Excluding overlapping and LTR duplicate sequences, our pool of potential HIV-1 targets contained **8, 846 unique** 19 mer sequences.

Gene	NL4-3 19mers	LANL 19mers	Virco 19mers *	LANL + V 19mers	19:19 Comparisons
LTR	616	235913	85889	321802	198230032
Gag	1485	2381419		2381419	3536407215
Pol	2994	4684143		4684143	14024324142
Vif	561	1407036	131805	1538841	863289801
Vpr	270	633152	131805	764957	206538390
Tat (x1)	197	642158	131805	773963	152470711
Rev (x1)	58	197213	131805	329018	19083044
Vpu	225	567764	131805	699569	157403025
Env	2547	9097846		9097846	23172213762
Tat (x2)	28	422240	85889	508129	14227612
Rev (x2)	257	1431363	85889	1517252	389933764
Nef	600	2943335	85889	3029224	1817534400
**Totals:**	**9838**			**Total 19mer to 19 mer comparisons:**	**44551655898**
**Unique:**	**8846**			**Total 19 mers:**	**24861276**

***** *The Virco data was generated as two groups of sequence for each sample. The first sequence covered the Vif, Vpr, Tat (exon 1), Rev (exon 1) and Vpu regions (105 sequences and 131805 19 mers), the second sequence covered the Tat (exon 2), Rev (exon 2), and Nef regions including a small amount of overlapping LTR sequence (83 sequences and 85889 19 mers). Each group was only counted once in the tally of total 19 mers analyzed (i.e. the sum of the LANL 19 mers + 131805 + 85889), rather than being counted 5 and 4 times respectively. However, all sequences in each group were compared to the corresponding NL4-3 19 mers and thus were included in the the tally of of total 19 mer : 19 mer comparisons.*

Conservation summary

8, 846 unique 19 mers from NL4-3 were compared against the 19 mers from the LANL and Virco HIV gene fragment sequence sets. The following table categorizes the conservation results, showing the number of targets and the percentage of the total 8, 846 NL4-3 19 mer targets that were in each category, for both all clades (LANL and Virco) and clade B only (LANL only).

	**LANL + Virco all clades**		**LANL subtype B**	
**Conservation**	**% of targets**	**# of targets**	**% of targets**	**# of targets**
100%	0.0	0	0.5	44
> 90 %	0.5	40	1.2	102
> 70 %	2.8	246	13.9	1227
> 50 %	10.4	921	37.1	3286
**Total number of 19 mer targets:**		**8846**		

The selected targets

The following table shows the **96** selected targets, along with sequence at the p -2, -1 and p +1, +2 positions, the loop sequence used, and a termination spacer base (**Tsp.**) if used. For targets that were made into a 20 bp hairpin, the last nucleotide of the loop was selected to be the complement of the last nucleotide of the p+2 position so that if the processed siRNA product(s) included the the last nucleotide of the loop then it too would be matched to the target. For targets made into a 21 bp hairpin, we used our default loop sequence (**A**CTCGAG**A**). The hairpins in which the last base of the anti-sense stem was 'T' also included a 'termination spacer' (**Tsp.**) so as to prevent premature termination via an early run of 'T's. This nucleotide was always the complement of the first nucleotide of the p-1 position (but never a 'T'), so that if included in the processed siRNA product(s) it was also matched to the target.

#	Target	p-2,1	Core 19 mer (p0)	p+1,2	Loop	Tsp.
1	LTR 63-20	CA	CAAGGCTACTTCCCTGATT	G**G**	**C**CTCGAG**C**	
2	LTR 506-20	AG	GGAACCCACTGCTTAAGCC	T**C**	**G**CTCGAG**G**	
3	LTR 507-20	GG	GAACCCACTGCTTAAGCCT	C**A**	**T**CTCGAG**T**	
4	LTR 509-20	GA	ACCCACTGCTTAAGCCTCA	A**A**	**A**CTCGAG**A**	
5	LTR 510-21	AA	CCCACTGCTTAAGCCTCAA	TA	**A**CTCGAG**A**	
6	LTR 516-21	CT	GCTTAAGCCTCAATAAAGC	TT	**A**CTCGAG**A**	
7	LTR 522-20	AA	GCCTCAATAAAGCTTGCCT	T**G**	**C**CTCGAG**C**	
8	LTR 527-21	T**C**	AATAAAGCTTGCCTTGAGT	GC	**A**CTCGAG**A**	G
9	LTR 553-20	AA	GTAGTGTGTGCCCGTCTGT	T**G**	**C**CTCGAG**C**	
10	Gag 532-20	AA	GGAGCCACCCCACAAGATT	T**A**	**T**CTCGAG**T**	
11	Gag 532-21	AA	GGAGCCACCCCACAAGATT	TA	**A**CTCGAG**A**	
12	Gag 533-20	AG	GAGCCACCCCACAAGATTT	A**A**	**T**CTCGAG**T**	
13	Gag 533-21	AG	GAGCCACCCCACAAGATTT	AA	**A**CTCGAG**A**	
14	Gag 534-20	G**G**	AGCCACCCCACAAGATTTA	A**A**	**T**CTCGAG**T**	C
15	Gag 534-21	G**G**	AGCCACCCCACAAGATTTA	AA	**A**CTCGAG**A**	C
16	Gag 535-20	GA	GCCACCCCACAAGATTTAA	A**T**	**A**CTCGAG**A**	
17	Gag 578-20	GG	GACATCAAGCAGCCATGCA	A**A**	**T**CTCGAG**T**	
18	Gag 698-20	GG	GAAGTGACATAGCAGGAAC	T**A**	**T**CTCGAG**T**	
19	Pol 242-20	A**G**	ATACAGGAGCAGATGATAC	A**G**	**C**CTCGAG**C**	C
20	Pol 246-21	A**C**	AGGAGCAGATGATACAGTA	TT	**A**CTCGAG**A**	G

#	Target	p-2,1	Core 19 mer (p0)	p+1,2	Loop	Tsp.
21	Pol 247-21	CA	GGAGCAGATGATACAGTAT	TA	**A**CTCGAG**A**	
22	Pol 248-20	AG	GAGCAGATGATACAGTATT	A**G**	**C**CTCGAG**C**	
23	Pol 248-21	AG	GAGCAGATGATACAGTATT	AG	**A**CTCGAG**A**	
24	Pol 249-20	G**G**	AGCAGATGATACAGTATTA	G**A**	**T**CTCGAG**T**	C
25	Pol 249-21	G**G**	AGCAGATGATACAGTATTA	GA	**A**CTCGAG**A**	C
26	Pol 250-20	GA	GCAGATGATACAGTATTAG	A**A**	**T**CTCGAG**T**	
27	Pol 250-21	GA	GCAGATGATACAGTATTAG	AA	**A**CTCGAG**A**	
28	Pol 251-20	AG	CAGATGATACAGTATTAGA	A**G**	**C**CTCGAG**C**	
29	Pol 404-20	AC	CTACACCTGTCAACATAAT	T**G**	**C**CTCGAG**C**	
30	Pol 529-21	T**T**	AAACAATGGCCATTGACAG	AA	**A**CTCGAG**A**	A
31	Pol 921-20	G**G**	ATGGAAAGGATCACCAGCA	A**T**	**A**CTCGAG**A**	C
32	Pol 925-20	G**G**	AAAGGATCACCAGCAATAT	T**C**	**G**CTCGAG**G**	C
33	Pol 926-20	GA	AAGGATCACCAGCAATATT	C**C**	**G**CTCGAG**G**	
34	Pol 1161-20	TA	TGAACTCCATCCTGATAAA	T**G**	**C**CTCGAG**C**	
35	Pol 2668-21	CA	GCAGTACAAATGGCAGTAT	TC	**A**CTCGAG**A**	
36	Pol 2670-21	G**C**	AGTACAAATGGCAGTATTC	AT	**A**CTCGAG**A**	G
37	Pol 2671-21	CA	GTACAAATGGCAGTATTCA	TC	**A**CTCGAG**A**	
38	Pol 2724-20	GG	GTACAGTGCAGGGGAAAGA	A**T**	**A**CTCGAG**A**	
39	Pol 2726-21	G**T**	ACAGTGCAGGGGAAAGAAT	AG	**A**CTCGAG**A**	A
40	Pol 2750-20	A**G**	ACATAATAGCAACAGACAT	A**C**	**G**CTCGAG**G**	C
41	Pol 2873-21	CT	GGAAAGGTGAAGGGGCAGT	AG	**A**CTCGAG**A**	
42	Pol 2874-21	TG	GAAAGGTGAAGGGGCAGTA	GT	**A**CTCGAG**A**	
43	Pol 2878-20	AA	GGTGAAGGGGCAGTAGTAA	T**A**	**T**CTCGAG**T**	
44	Pol 2878-21	AA	GGTGAAGGGGCAGTAGTAA	TA	**A**CTCGAG**A**	
45	Pol 2879-20	AG	GTGAAGGGGCAGTAGTAAT	A**C**	**G**CTCGAG**G**	
46	Pol 2879-21	AG	GTGAAGGGGCAGTAGTAAT	AC	**A**CTCGAG**A**	
47	Pol 2880-20	GG	TGAAGGGGCAGTAGTAATA	C**A**	**T**CTCGAG**T**	
48	Pol 2881-21	GT	GAAGGGGCAGTAGTAATAC	AA	**A**CTCGAG**A**	
49	Pol 2882-21	T**G**	AAGGGGCAGTAGTAATACA	AG	**A**CTCGAG**A**	C
50	Pol 2885-20	AG	GGGCAGTAGTAATACAAGA	T**A**	**T**CTCGAG**T**	
51	Vif 9-21	AA	CAGATGGCAGGTGATGATT	GT	**A**CTCGAG**A**	
52	Vif 10-21	A**C**	AGATGGCAGGTGATGATTG	TG	**A**CTCGAG**A**	G
53	Vif 11-21	CA	GATGGCAGGTGATGATTGT	GT	**A**CTCGAG**A**	

#	Target	p-2,1	Core 19 mer (p0)	p+1,2	Loop	Tsp.
54	Vif 12-20	A**G**	ATGGCAGGTGATGATTGTG	T**G**	**C**CTCGAG**C**	C
55	Vif 14-21	AT	GGCAGGTGATGATTGTGTG	GC	**A**CTCGAG**A**	
56	Vif 15-21	TG	GCAGGTGATGATTGTGTGG	CA	**A**CTCGAG**A**	
57	Vif 37-20	AA	GTAGACAGGATGAGGATTA	A**C**	**G**CTCGAG**G**	
58	Tat (x1) 131-20	AG	GCATCTCCTATGGCAGGAA	G**A**	**T**CTCGAG**T**	
59	Tat (x1) 132-21	GG	CATCTCCTATGGCAGGAAG	AA	**A**CTCGAG**A**	
60	Tat (x1) 140-21	C**T**	ATGGCAGGAAGAAGCGGAG	AC	**A**CTCGAG**A**	A
61	Tat (x1) 143-21	TG	GCAGGAAGAAGCGGAGACA	GC	**A**CTCGAG**A**	
62	Tat (x1) 147-20	AG	GAAGAAGCGGAGACAGCGA	C**G**	**C**CTCGAG**C**	
63	Rev (x1) 9-20	G**G**	AAGAAGCGGAGACAGCGAC	G**A**	**T**CTCGAG**T**	C
64	Vpu 139-21	TA	GAAAGAGCAGAAGACAGTG	GC	**A**CTCGAG**A**	
65	Vpu 140-20	A**G**	AAAGAGCAGAAGACAGTGG	C**A**	**T**CTCGAG**T**	C
66	Vpu 140-21	A**G**	AAAGAGCAGAAGACAGTGG	CA	**A**CTCGAG**A**	C
67	Vpu 141-20	GA	AAGAGCAGAAGACAGTGGC	A**A**	**T**CTCGAG**T**	
68	Vpu 143-20	AA	GAGCAGAAGACAGTGGCAA	T**G**	**C**CTCGAG**C**	
69	Vpu 144-20	A**G**	AGCAGAAGACAGTGGCAAT	G**A**	**T**CTCGAG**T**	C
70	Vpu 144-21	A**G**	AGCAGAAGACAGTGGCAAT	GA	**A**CTCGAG**A**	C
71	Vpu 145-20	GA	GCAGAAGACAGTGGCAATG	A**G**	**C**CTCGAG**C**	
72	Vpu 146-20	AG	CAGAAGACAGTGGCAATGA	G**A**	**T**CTCGAG**T**	
73	Vpu 147-21	G**C**	AGAAGACAGTGGCAATGAG	AG	**A**CTCGAG**A**	G
74	Vpu 148-21	CA	GAAGACAGTGGCAATGAGA	GT	**A**CTCGAG**A**	
75	Vpu 149-20	A**G**	AAGACAGTGGCAATGAGAG	T**G**	**C**CTCGAG**C**	C
76	Env 628-20	AG	CCAATTCCCATACATTATT	G**T**	**A**CTCGAG**A**	
77	Env 1423-20	GG	GACAATTGGAGAAGTGAAT	T**A**	**T**CTCGAG**T**	
78	Env 1423-21	GG	GACAATTGGAGAAGTGAAT	TA	**A**CTCGAG**A**	
79	Env 1424-21	G**G**	ACAATTGGAGAAGTGAATT	AT	**A**CTCGAG**A**	C
80	Env 1425-20	GA	CAATTGGAGAAGTGAATTA	T**A**	**T**CTCGAG**T**	
81	Env 1425-21	GA	CAATTGGAGAAGTGAATTA	TA	**A**CTCGAG**A**	
82	Env 1426-21	A**C**	AATTGGAGAAGTGAATTAT	AT	**A**CTCGAG**A**	G
83	Env 1428-21	AA	TTGGAGAAGTGAATTATAT	AA	**A**CTCGAG**A**	
84	Env 1431-21	TG	GAGAAGTGAATTATATAAA	TA	**A**CTCGAG**A**	
85	Env 1432-20	G**G**	AGAAGTGAATTATATAAAT	A**T**	**A**CTCGAG**A**	C
86	Env 1432-21	G**G**	AGAAGTGAATTATATAAAT	AT	**A**CTCGAG**A**	C

#	Target	p-2,1	Core 19 mer (p0)	p+1,2	Loop	Tsp.
87	Env 1433-20	GA	GAAGTGAATTATATAAATA	T**A**	**T**CTCGAG**T**	
88	Env 1433-21	GA	GAAGTGAATTATATAAATA	TA	**A**CTCGAG**A**	
89	Env 1434-20	A**G**	AAGTGAATTATATAAATAT	A**A**	**T**CTCGAG**T**	C
90	Env 1436-20	AA	GTGAATTATATAAATATAA	A**G**	**C**CTCGAG**C**	
91	Env 1437-20	AG	TGAATTATATAAATATAAA	G**T**	**A**CTCGAG**A**	
92	Env 2308-20	T**G**	AGAGACTTACTCTTGATTG	T**A**	**T**CTCGAG**T**	C
93	Rev (x2) 158-20	CT	TGAGAGACTTACTCTTGAT	T**G**	**C**CTCGAG**C**	
94	Rev (x2) 159-20	TT	GAGAGACTTACTCTTGATT	G**T**	**A**CTCGAG**A**	
95	Nef 350-20	CA	CACAAGGCTACTTCCCTGA	T**T**	**A**CTCGAG**A**	
96	Nef 351-20	A**C**	ACAAGGCTACTTCCCTGAT	T**G**	**C**CTCGAG**C**	G

n.b. The target positions are the co-ordinates within each gene of NL4-3.

The 22 regions

The following table shows the regions # (**R**), the shRNA # (**hp #**), the number of shRNA within each region (**T**), the starting co-ordinates of the first and last target within each region (and the specific gene product area if applicable), whether or not the region was unique amongst all previously made (**U14**: 14 regions were unique) or previously proposed (**U11**: 11 regions were unique) targets (see **Additional file 1** and Naito *et. at.* (2007)), and the sequence of the region spanning the first nucleotide of the first p0 19 mer target core to the last nucleotide of the last p0 19 mer target core within each region. n.b. the common regions cover the overlapping 19 nt. cores only, i.e. some of the first nucleotides of the p-2 and p-1 positions, and some of the final nucleotides of the p+1 and p+2 positions are not covered (see the individual core data after for these).

R	hp #	T	Target	U14	U11	Sequence region
1	1, 95-96	3	LTR 63 (U3) / Nef 350-52	U	U	CACAAGGCTACTTCCCTGATTG
2	2-8.	7	LTR 506-27 (R)			GGAACCCACTGCTTAAGCCTCAATAAAGCTTGCCTT-GAGTGC
3	9	1	LTR 553 (U5)	U		GTAGTGTGTGCCCGTCTGTT
4	10-16.	7	Gag 532-35 (P24: CA)	U	U	GGAGCCACCCCACAAGATTTAAA
5	17	1	Gag 578 (P24: CA)	U	U	GACATCAAGCAGCCATGCAA
6	18	1	Gag 698 (P24: CA)	U	U	GAAGTGACATAGCAGGAACT
7	19-28	10	Pol 242-51			ATACAGGAGCAGATGATACAGTATTAGAA
8	29	1	Pol 404	U		CTACACCTGTCAACATAATT
9	30	1	Pol 529 (P51: RT)	U		AAACAATGGCCATTGACAGAA
10	31-33	3	Pol 921-26 (P51: RT)			ATGGAAAGGATCACCAGCAATATTC
11	34	1	Pol 1161 (P51: RT)	U	U	TGAACTCCATCCTGATAAAT
12	35-37	3	Pol 2668-71 (P31: IN)			GCAGTACAAATGGCAGTATTCATC
13	38-39	2	Pol 2724-26 (P31: IN)			GTACAGTGCAGGGGAAAGAATAG
14	40	1	Pol 2750 (P31: IN)	U	U	ACATAATAGCAACAGACATA
15	41-50	10	Pol 2873-85 (P31: IN)			GGAAAGGTGAAGGGGCAGTAGTAATACAAGAT
16	51-56	6	Vif 9-15 (P31: IN, Vif)	U	U	CAGATGGCAGGTGATGATTGTGTGGCA
17	57	1	Vif 37 (P31: IN, Vif-xj)	U	U	GTAGACAGGATGAGGATTAA
18	58-63	6	Tat (x1) 131-43 / Rev (x1) 8-9	U	U	GCATCTCCTATGGCAGGAAGAAGCGGAGACAG-CGACG
19	64-77	12	Vpu 139-49			GAAAGAGCAGAAGACAGTGGCAATGAGAGT
20	76	1	Env 628 (GP120)	U	U	CCAATTCCCATACATTATTG

R	hp #	T	Target	U14	U11	Sequence region
21	77-91	15	Env 1423-37 (GP120)			GACAATTGGAGAAGTGAATTATATAAATATAAAG
22	92-94	4	Env 2308 (GP41) / Rev (x2) 158-60	U	U	TGAGAGACTTACTCTTGATTGTGT

n.b. CA: Capsid, RT: Reverse Transcriptase, IN: Integrase, x1: exon1, x2: exon2, xj: crosses and ORF junction.

U14: These are the 14 regions that are unique amongst all previously made and published si- and shRNAs.

U11: These are the 11 regions that are unique amongst all previously proposed targets.

Matched target pairs

The 96 hairpins were comprised of 53 hairpins with 20 bp stems, and 43 hairpins with 21 bp stems, including **14** matched pairs of hairpins with 20 and 21 bp stems designed to the same target site. The pairs are listed in the following with the 3 replicate fluorescence values (as a % of the control) from the fluorescent reporter assays used for statistical analysis shown.

	First shRNA		Replicate % F.			Second shRNA		Replicate % F.		
**Pair**	**#**	**Target**	**r1**	**r2**	**r3**	**#**	**Target**	**r1**	**r2**	**r3**
**1**	10	Gag 532-20	57.9	52.1	59.9	11	Gag 532-21	41.3	36.2	51.4
**2**	12	Gag 533-20	30.7	23.8	29.4	13	Gag 533-21	31.6	27.9	39.1
**3**	14	Gag 534-20	46.5	36.8	40.1	15	Gag 534-21	43.7	41.9	49.1
**4**	22	Pol 248-20	9.0	7.8	9.7	23	Pol 248-21	9.1	7.9	9.1
**5**	24	Pol 249-20	10.1	7.9	9.5	25	Pol 249-21	9.0	8.0	9.1
**6**	26	Pol 250-20	11.2	8.6	9.6	27	Pol 250-21	10.3	8.6	10.3
**7**	43	Pol 2878-20	7.9	8.0	7.2	44	Pol 2878-21	8.6	7.2	8.2
**8**	45	Pol 2879-20	8.4	8.1	7.9	46	Pol 2879-21	99.6	95.6	93.4
**9**	65	Vpu 140-20	34.2	27.1	30.4	66	Vpu 140-21	27.2	23.0	23.9
**10**	69	Vpu 144-20	7.4	5.8	7.0	70	Vpu 144-21	6.5	5.6	5.8
**11**	77	Env 1423-20	6.1	5.8	6.4	78	Env 1423-21	8.1	7.4	9.5
**12**	80	Env 1425-20	12.3	10.4	11.4	81	Env 1425-21	7.5	6.8	7.3
**13**	85	Env 1432-20	12.7	11.8	11.4	86	Env 1432-21	7.2	6.6	7.9
**14**	87	Env 1433-20	19.3	17.2	18.1	88	Env 1433-21	19.0	18.4	19.3

Overlapping target pairs

The **19** regions of overlapping hairpins comprised **73** adjacent pairs of hairpins where the starting position of the targets differed by 1 nt. **Forty two** of these had the same stem length (either both 20 or both 21 bp), **31** had different stem lengths. The pairs are listed in the following 2 tables grouped according to pairs with the same, and pairs with different stem lengths. The 3 replicate fluorescence values (as a % of the control) from the fluorescent reporter assays used for statistical analysis are shown.

The 42 pairs with matched stem lengths

	First shRNA		Replicate % F.			Second shRNA		Replicate % F.		
**Pair**	**#**	**Target**	**r1**	**r2**	**r3**	**#**	**Target**	**r1**	**r2**	**r3**
**1**	2	Ltr 506-20	66.7	82.4	68.6	3	Ltr 507-20	73.6	85.9	77.5
**2**	10	Gag 532-20	57.86	52.08	59.92	12	Gag 533-20	30.7	23.8	29.4
**3**	11	Gag 532-21	41.3	36.2	51.4	13	Gag 533-21	31.6	27.9	39.1
**4**	12	Gag 533-20	30.69	23.82	29.39	14	Gag 534-20	46.5	36.8	40.1
**5**	13	Gag 533-21	31.6	27.9	39.1	15	Gag 534-21	43.7	41.9	49.1
**6**	14	Gag 534-20	46.5	36.8	40.1	16	Gag 535-20	42.7	29.6	34.8
**7**	20	Pol 0246-21	9.1	7.6	8.5	21	Pol 0247-21	10.0	9.3	10.6
**8**	21	Pol 0247-21	10.0	9.3	10.6	23	Pol 0248-21	9.1	7.9	9.1
**9**	22	Pol 0248-20	9.0	7.8	9.7	24	Pol 0249-20	10.1	7.9	9.5
**10**	23	Pol 0248-21	9.1	7.9	9.1	25	Pol 0249-21	9.0	8.0	9.1
**11**	24	Pol 0249-20	10.1	7.9	9.5	26	Pol 0250-20	11.2	8.6	9.6
**12**	25	Pol 0249-21	9.0	8.0	9.1	27	Pol 0250-21	10.3	8.6	10.3
**13**	26	Pol 0250-20	11.2	8.6	9.6	28	Pol 0251-20	119	120.9	135.2
**14**	32	Pol 0925-20	14.1	15.0	14.6	33	Pol 0926-20	17.3	23.3	21.6
**15**	36	Pol 2670-21	9.9	9.3	9.4	37	Pol 2671-21	10.6	9.3	10.0
**16**	41	Pol 2873-21	38.3	40.4	39.5	42	Pol 2874-21	23.2	19.9	18.2
**17**	43	Pol 2878-20	7.9	8.0	7.2	45	Pol 2879-20	8.4	8.1	7.9
**18**	44	Pol 2878-21	8.6	7.2	8.2	46	Pol 2879-21	99.6	95.6	93.4
**19**	45	Pol 2879-20	8.4	8.1	7.9	47	Pol 2880-20	29.9	30.9	27.7
**20**	48	Pol 2881-21	12.2	12.4	11.8	49	Pol 2882-21	9.6	8.6	8.3
**21**	51	Vif 9-21	16.6	16.9	14.5	52	Vif 10-21	13.9	12.8	12.0
**22**	52	Vif 10-21	13.9	12.8	12.0	53	Vif 11-21	14.2	14.3	13.5

	First shRNA		Replicate % F.			Second shRNA		Replicate % F.		
**Pair**	**#**	**Target**	**r1**	**r2**	**r3**	**#**	**Target**	**r1**	**r2**	**r3**
**23**	55	Vif 14-21	16.8	17.5	15.8	56	Vif 15-21	11.6	11.1	10.5
**24**	62	Revx1 8-20	18.7	16.4	14.9	63	Revx1 9-20	15.9	14.6	14.4
**25**	64	Vpu 139-21	10.4	7.5	9.2	66	Vpu 140-21	27.2	23.0	23.9
**26**	65	Vpu 140-20	34.2	27.1	30.4	67	Vpu 141-20	7.1	5.7	6.8
**27**	68	Vpu 143-20	7.0	6.1	6.9	69	Vpu 144-20	7.4	5.8	7.0
**28**	69	Vpu 144-20	7.4	5.8	7.0	71	Vpu 145-20	7.6	5.8	7.1
**29**	71	Vpu 145-20	7.6	5.8	7.1	72	Vpu 146-20	8.6	6.1	7.3
**30**	73	Vpu 147-21	6.5	5.7	6.4	74	Vpu 148-21	6.7	5.3	6.0
**31**	78	Env 1423-21	8.1	7.4	9.5	79	Env 1424-21	6.5	6.1	7.0
**32**	79	Env 1424-21	6.5	6.1	7.0	81	Env 1425-21	7.5	6.8	7.3
**33**	81	Env 1425-21	7.5	6.8	7.3	82	Env 1426-21	7.6	6.3	7.0
**34**	84	Env 1431-21	32.2	22.9	29.2	86	Env 1432-21	7.2	6.6	7.9
**35**	85	Env 1432-20	12.7	11.8	11.4	87	Env 1433-20	19.3	17.2	18.1
**36**	86	Env 1432-21	7.2	6.6	7.9	88	Env 1433-21	19.0	18.4	19.3
**37**	87	Env 1433-20	19.3	17.2	18.1	89	Env 1434-20	20.9	18.6	20.3
**38**	90	Env 1436-20	44.0	41.3	41.4	91	Env 1437-20	64.9	62.3	61.9
**39**	93	Revx2 158-20	7.8	7.7	8.4	94	Revx2 159-20	11.3	9.9	12.7
**40**	94	Revx2 159-20	11.3	9.9	12.7	92	Env 2308-20	20.3	17.1	18.8
**41**	95	Nef 350-20	29.6	25.5	28.6	96	Nef 351-20	37.7	38.9	38.6
**42**	96	Nef 351-20	37.7	38.9	38.6	1	Ltr 63-20	57.2	57.5	54.2

The 31 pairs with mismatched stem lengths

	First shRNA		Replicate % F.			Second shRNA		Replicate % F.		
**Pair**	**#**	**Target**	**r1**	**r2**	**r3**	**#**	**Target**	**r1**	**r2**	**r3**
**1**	4	Ltr 509-20	39.3	35.6	32.7	5	Ltr 510-21	17.3	17.7	21.5
**2**	10	Gag 532-20	57.86	52.08	59.92	13	Gag 533-21	31.6	27.9	39.1
**3**	11	Gag 532-21	41.3	36.2	51.4	12	Gag 533-20	30.7	23.8	29.4
**4**	12	Gag 533-20	30.7	23.8	29.4	15	Gag 534-21	43.7	41.9	49.1
**5**	13	Gag 533-21	31.6	27.9	39.1	14	Gag 534-20	46.5	36.8	40.1
**6**	15	Gag 534-21	43.7	41.9	49.1	16	Gag 535-20	42.7	29.6	34.8
**7**	21	Pol 0247-21	10.0	9.3	10.6	22	Pol 0248-20	9.0	7.8	9.7
**8**	22	Pol 0248-20	9.0	7.8	9.7	25	Pol 0249-21	9.0	8.0	9.1

	First shRNA		Replicate % F.			Second shRNA		Replicate % F.		
**Pair**	**#**	**Target**	**r1**	**r2**	**r3**	**#**	**Target**	**r1**	**r2**	**r3**
**9**	23	Pol 0248-21	9.1	7.9	9.1	24	Pol 0249-20	10.1	7.9	9.5
**10**	24	Pol 0249-20	10.1	7.9	9.5	27	Pol 0250-21	10.3	8.6	10.3
**11**	25	Pol 0249-21	9.0	8.0	9.1	26	Pol 0250-20	11.2	8.6	9.6
**12**	27	Pol 0250-21	10.3	8.6	10.3	28	Pol 0251-20	119	120.9	135.2
**13**	43	Pol 2878-20	7.9	8.0	7.2	46	Pol 2879-21	99.6	95.6	93.4
**14**	44	Pol 2878-21	8.6	7.2	8.2	45	Pol 2879-20	8.4	8.1	7.9
**15**	46	Pol 2879-21	99.6	95.6	93.4	47	Pol 2880-20	29.9	30.9	27.7
**16**	47	Pol 2880-20	29.9	30.9	27.7	48	Pol 2881-21	12.2	12.4	11.8
**17**	53	Vif 11-21	14.2	14.3	13.5	54	Vif 12-20	20.5	25.3	22.8
**18**	58	Tatx1 131-20	28.8	24.5	25.2	59	Tatx1 132-21	28.2	27.8	24.4
**19**	64	Vpu 139-21	10.4	7.5	9.2	65	Vpu 140-20	34.2	27.1	30.4
**20**	66	Vpu 140-21	27.2	23.0	23.9	67	Vpu 141-20	7.1	5.7	6.8
**21**	68	Vpu 143-20	7.0	6.1	6.9	70	Vpu 144-21	6.5	5.6	5.8
**22**	70	Vpu 144-21	6.5	5.6	5.8	71	Vpu 145-20	7.6	5.8	7.1
**23**	72	Vpu 146-20	8.6	6.1	7.3	73	Vpu 147-21	6.5	5.7	6.4
**24**	74	Vpu 148-21	6.7	5.3	6.0	75	Vpu 149-20	8.9	6.8	7.6
**25**	77	Env 1423-20	6.1	5.8	6.4	79	Env 1424-21	6.5	6.1	7.0
**26**	79	Env 1424-21	6.5	6.1	7.0	80	Env 1425-20	12.3	10.4	11.4
**27**	80	Env 1425-20	12.3	10.4	11.4	82	Env 1426-21	7.6	6.3	7.0
**28**	84	Env 1431-21	32.2	22.9	29.2	85	Env 1432-20	12.7	11.8	11.4
**29**	85	Env 1432-20	12.7	11.8	11.4	88	Env 1433-21	19.0	18.4	19.3
**30**	86	Env 1432-21	7.2	6.6	7.9	87	Env 1433-20	19.3	17.2	18.1
**31**	88	Env 1433-21	19.0	18.4	19.3	89	Env 1434-20	20.9	18.6	20.3

Detailed conservation profiles

The following table shows the detailed conservation profiles for the **96** selected targets. Conservations were calculated for LANL and Virco sequences combined, LANL clade B subtypes only, and the Virco sequences only (where applicable). In each group, 6 conservations are given: the conservation for the entire overlapping 23 mer region, and the 5 individual conservations for the 5 overlapping 19 mers that make up the 23 mer profile region. Some of shRNAs were matched to more than 1 gene, however, conservations were only estimated using a single gene data set (corresponding to the shRNA / target name), e.g. hairpin cores 1, 95, and 96 were slightly higher conserved in the Nef data set rather than the LTR data set (LTR conservations shown).

		LANL + Virco (all)						LANL clade B						Virco (all)					
**#**	**Target**	**23**	**-2**	**-1**	**p0**	**+1**	**+2**	**23**	**-2**	**-1**	**p0**	**+1**	**+2**	**23**	**-2**	**-1**	**p0**	**+1**	**+2**
1	LTR 63-20	32	34	32	**33**	33	33	73	78	75	**76**	75	75	39	44	39	**40**	40	40
2	LTR 506-20	50	51	64	**67**	69	69	97	97	97	**97**	98	100	-	-	-	**-**	-	-
3	LTR 507-20	64	64	67	**69**	69	69	97	97	97	**98**	100	100	-	-	-	**-**	-	-
4	LTR 509-20	69	69	69	**69**	70	71	98	98	100	**100**	100	100	-	-	-	**-**	-	-
5	LTR 510-21	69	69	69	**70**	71	71	100	100	100	**100**	100	100	-	-	-	**-**	-	-
6	LTR 516-21	71	71	71	**71**	71	71	100	100	100	**100**	100	100	-	-	-	**-**	-	-
7	LTR 522-20	90	93	91	**92**	92	93	100	100	100	**100**	100	100	-	-	-	**-**	-	-
8	LTR 527-21	90	93	93	**93**	93	91	100	100	100	**100**	100	100	-	-	-	**-**	-	-
9	LTR 553-20	48	51	52	**51**	50	50	90	94	92	**92**	92	90	-	-	-	**-**	-	-
10	Gag 532-20	58	64	62	**70**	70	69	77	79	78	**80**	80	80	-	-	-	**-**	-	-
11	Gag 532-21	58	64	62	**70**	70	69	77	79	78	**80**	80	80	-	-	-	**-**	-	-
12	Gag 533-20	58	62	70	**70**	69	71	77	78	80	**80**	80	82	-	-	-	**-**	-	-
13	Gag 533-21	58	62	70	**70**	69	71	77	78	80	**80**	80	82	-	-	-	**-**	-	-
14	Gag 534-20	68	70	70	**69**	71	71	79	80	80	**80**	82	82	-	-	-	**-**	-	-
15	Gag 534-21	68	70	70	**69**	71	71	79	80	80	**80**	82	82	-	-	-	**-**	-	-
16	Gag 535-20	16	70	69	**71**	71	16	6	80	80	**82**	82	6	-	-	-	**-**	-	-
17	Gag 578-20	55	62	63	**63**	57	58	72	78	79	**79**	73	73	-	-	-	**-**	-	-
18	Gag 698-20	67	74	71	**70**	68	69	74	78	78	**76**	75	75	-	-	-	**-**	-	-
19	Pol 242-20	57	58	58	**58**	57	83	76	77	77	**77**	77	84	-	-	-	**-**	-	-
20	Pol 246-21	76	83	83	**80**	80	80	78	84	84	**81**	87	87	-	-	-	**-**	-	-
21	Pol 247-21	75	83	80	**80**	80	79	77	84	81	**87**	87	86	-	-	-	**-**	-	-

		LANL + Virco (all)						LANL clade B						Virco (all)					
**#**	**Target**	**23**	**-2**	**-1**	**p0**	**+1**	**+2**	**23**	**-2**	**-1**	**p0**	**+1**	**+2**	**23**	**-2**	**-1**	**p0**	**+1**	**+2**
22	Pol 248-20	75	80	80	**80**	79	80	77	81	87	**87**	86	87	-	-	-	**-**	-	-
23	Pol 248-21	75	80	80	**80**	79	80	77	81	87	**87**	86	87	-	-	-	**-**	-	-
24	Pol 249-20	78	80	80	**79**	80	80	85	87	87	**86**	87	87	-	-	-	**-**	-	-
25	Pol 249-21	78	80	80	**79**	80	80	85	87	87	**86**	87	87	-	-	-	**-**	-	-
26	Pol 250-20	77	80	79	**80**	80	79	85	87	86	**87**	87	87	-	-	-	**-**	-	-
27	Pol 250-21	77	80	79	**80**	80	79	85	87	86	**87**	87	87	-	-	-	**-**	-	-
28	Pol 251-20	75	79	80	**80**	79	78	85	86	87	**87**	87	86	-	-	-	**-**	-	-
29	Pol 404-20	57	58	67	**67**	67	70	64	64	65	**65**	65	68	-	-	-	**-**	-	-
30	Pol 529-21	62	64	64	**68**	68	66	78	80	80	**84**	84	81	-	-	-	**-**	-	-
31	Pol 921-20	58	60	60	**58**	61	61	75	78	78	**76**	79	78	-	-	-	**-**	-	-
32	Pol 925-20	50	61	60	**60**	60	51	72	78	77	**77**	77	73	-	-	-	**-**	-	-
33	Pol 926-20	50	60	60	**60**	51	53	72	77	77	**77**	73	74	-	-	-	**-**	-	-
34	Pol 1161-20	32	36	34	**32**	32	32	75	77	77	**75**	75	76	-	-	-	**-**	-	-
35	Pol 2668-21	72	82	79	**78**	78	73	73	87	85	**84**	84	74	-	-	-	**-**	-	-
36	Pol 2670-21	72	78	78	**73**	73	74	73	84	84	**74**	74	74	-	-	-	**-**	-	-
37	Pol 2671-21	22	78	73	**73**	74	22	57	84	74	**74**	74	57	-	-	-	**-**	-	-
38	Pol 2724-20	69	70	71	**71**	81	81	62	63	63	**67**	86	86	-	-	-	**-**	-	-
39	Pol 2726-21	14	71	81	**81**	79	18	41	67	86	**86**	82	50	-	-	-	**-**	-	-
40	Pol 2750-20	65	66	65	**65**	65	70	74	75	75	**75**	74	77	-	-	-	**-**	-	-
41	Pol 2873-21	77	82	88	**88**	85	84	80	84	95	**95**	93	91	-	-	-	**-**	-	-
42	Pol 2874-21	84	88	88	**85**	84	84	91	95	95	**93**	91	91	-	-	-	**-**	-	-
43	Pol 2878-20	74	84	76	**74**	74	74	79	91	80	**80**	79	79	-	-	-	**-**	-	-
44	Pol 2878-21	74	84	76	**74**	74	74	79	91	80	**80**	79	79	-	-	-	**-**	-	-
45	Pol 2879-20	73	76	74	**74**	74	74	79	80	80	**79**	79	80	-	-	-	**-**	-	-
46	Pol 2879-21	73	76	74	**74**	74	74	79	80	80	**79**	79	80	-	-	-	**-**	-	-
47	Pol 2880-20	74	74	74	**74**	74	74	79	80	79	**79**	80	80	-	-	-	**-**	-	-
48	Pol 2881-21	63	74	74	**74**	74	64	78	79	79	**80**	80	79	-	-	-	**-**	-	-
49	Pol 2882-21	63	74	74	**74**	64	64	78	79	80	**80**	79	79	-	-	-	**-**	-	-
50	Pol 2885-20	39	64	64	**64**	40	42	67	79	79	**79**	67	68	-	-	-	**-**	-	-
51	Vif 9-21	71	71	71	**71**	71	71	91	91	91	**92**	92	92	74	75	75	**74**	74	74
52	Vif 10-21	69	71	71	**71**	71	70	88	91	92	**92**	92	89	74	75	74	**74**	74	75
53	Vif 11-21	69	71	71	**71**	70	70	88	92	92	**92**	89	90	74	74	74	**74**	75	76
54	Vif 12-20	69	71	71	**70**	70	70	88	92	92	**89**	90	90	74	74	74	**75**	76	76
55	Vif 14-21	69	70	70	**70**	70	70	89	89	90	**90**	90	90	75	75	76	**76**	76	76

#	Target	LANL + Virco (all)						LANL clade B						Virco (all)					
		23	-2	-1	p0	+1	+2	23	-2	-1	p0	+1	+2	23	-2	-1	p0	+1	+2
56	Vif 15-21	70	70	70	**70**	70	70	89	90	90	**90**	90	90	76	76	76	**76**	76	76
57	Vif 37-20	2	45	43	**71**	13	4	3	76	75	**87**	17	6	2	36	35	**67**	18	3
58	Tat (x1) 131-20	68	68	68	**68**	68	70	83	84	84	**84**	84	85	57	58	58	**57**	57	57
59	Tat (x1) 132-21	68	68	68	**68**	70	70	84	84	84	**84**	85	86	57	58	57	**57**	57	58
60	Tat (x1) 140-21	68	81	82	**73**	72	72	73	79	78	**77**	75	75	62	77	80	**77**	74	73
61	Tat (x1) 143-21	60	72	68	**62**	60	60	68	75	74	**70**	68	68	57	73	66	**58**	58	57
62	Tat (x1) 147-20	59	60	60	**60**	59	59	67	68	68	**68**	67	67	54	58	57	**57**	56	54
63	Rev (x1) 9-20	57	61	60	**60**	59	58	66	68	68	**68**	67	66	52	57	57	**56**	54	53
64	Vpu 139-21	6	6	44	**64**	64	56	12	12	70	**77**	78	72	6	6	49	**76**	76	66
65	Vpu 140-20	36	44	64	**64**	56	65	65	70	77	**78**	72	82	40	49	76	**76**	66	69
66	Vpu 140-21	36	44	64	**64**	56	65	65	70	77	**78**	72	82	40	49	76	**76**	66	69
67	Vpu 141-20	55	64	64	**56**	65	66	72	77	78	**72**	82	83	66	76	76	**66**	69	69
68	Vpu 143-20	55	56	65	**66**	65	66	72	72	82	**83**	83	83	65	66	69	**69**	68	69
69	Vpu 144-20	65	65	66	**65**	66	67	82	82	83	**83**	83	84	68	69	69	**68**	69	73
70	Vpu 144-21	65	65	66	**65**	66	67	82	82	83	**83**	83	84	68	69	69	**68**	69	73
71	Vpu 145-20	49	66	65	**66**	67	53	60	83	83	**83**	84	63	56	69	68	**69**	73	61
72	Vpu 146-20	48	65	66	**67**	53	53	59	83	83	**84**	63	62	56	68	69	**73**	61	61
73	Vpu 147-21	50	66	67	**53**	53	53	60	83	84	**63**	62	62	56	69	73	**61**	61	61
74	Vpu 148-21	46	67	53	**53**	53	49	55	84	63	**62**	62	56	48	73	61	**61**	61	50
75	Vpu 149-20	48	53	53	**53**	49	49	56	63	62	**62**	56	57	50	61	61	**61**	50	53
76	Env 628-20	45	46	46	**61**	61	60	63	64	65	**85**	85	84	-	-	-	**-**	-	-
77	Env 1423-20	48	53	67	**72**	74	74	60	66	73	**76**	77	77	-	-	-	**-**	-	-
78	Env 1423-21	48	53	67	**72**	74	74	60	66	73	**76**	77	77	-	-	-	**-**	-	-
79	Env 1424-21	63	67	72	**74**	74	79	70	73	76	**77**	77	84	-	-	-	**-**	-	-
80	Env 1425-20	71	72	74	**74**	79	79	75	76	77	**77**	84	84	-	-	-	**-**	-	-
81	Env 1425-21	71	72	74	**74**	79	79	75	76	77	**77**	84	84	-	-	-	**-**	-	-
82	Env 1426-21	67	74	74	**79**	79	73	75	77	77	**84**	84	81	-	-	-	**-**	-	-
83	Env 1428-21	72	79	79	**73**	75	75	80	84	84	**81**	82	82	-	-	-	**-**	-	-
84	Env 1431-21	65	75	75	**65**	65	65	80	82	82	**81**	81	81	-	-	-	**-**	-	-
85	Env 1432-20	62	75	65	**65**	65	63	78	82	81	**81**	81	79	-	-	-	**-**	-	-
86	Env 1432-21	62	75	65	**65**	65	63	78	82	81	**81**	81	79	-	-	-	**-**	-	-
87	Env 1433-20	62	65	65	**65**	63	64	78	81	81	**81**	79	80	-	-	-	**-**	-	-
88	Env 1433-21	62	65	65	**65**	63	64	78	81	81	**81**	79	80	-	-	-	**-**	-	-
89	Env 1434-20	62	65	65	**63**	64	64	78	81	81	**79**	80	80	-	-	-	**-**	-	-

		LANL + Virco (all)						LANL clade B						Virco (all)					
**#**	**Target**	**23**	**-2**	**-1**	**p0**	**+1**	**+2**	**23**	**-2**	**-1**	**p0**	**+1**	**+2**	**23**	**-2**	**-1**	**p0**	**+1**	**+2**
90	Env 1436-20	61	63	64	**64**	66	66	78	79	80	**80**	83	83	-	-	-	**-**	-	-
91	Env 1437-20	62	64	64	**66**	66	67	79	80	80	**83**	83	83	-	-	-	**-**	-	-
92	Env 2308-20	27	44	43	**43**	37	28	48	76	75	**74**	66	49	-	-	-	**-**	-	-
93	Rev (x2) 158-20	44	47	49	**48**	48	46	69	74	77	**76**	75	72	32	33	38	**38**	38	37
94	Rev (x2) 159-20	39	49	48	**48**	46	40	64	77	76	**75**	72	65	21	38	38	**38**	37	21
95	Nef 350-20	47	50	51	**51**	49	49	60	63	64	**64**	62	62	36	40	44	**44**	39	40
96	Nef 351-20	48	51	51	**49**	49	49	61	64	64	**62**	62	62	39	44	44	**39**	40	40
	**Average 96:**	59	66	68	68	67	65	74	80	81	81	80	78	52	60	63	63	60	59
	**Virco equiv. *:**	52	59	61	62	59	58	68	77	79	78	74	73	52	60	63	63	60	59

***** *Virco equiv. = Virco equivalent sequences*

Reporter lengths and average activities

Two groups of fluorescent reporters were used in this study. The 'original' reporters made up of GFP fusions to each of the full length HIV genes. The accessory gene reporters were classified as short reporters given their relative shorter lengths compared to the the 3 core genes, which were classified as long reporters. A second group of reporters was for the long core reporters, consisting of shorter fragments spanning overlapping regions of each core gene and containing only that sequence targeted. The following table summarizes each reporter including its length and the average % fluorescence (**A. %F.**) of all the shRNAs measured with each reporter. Also shown for the second group of shortened core reporters is the original average % fluorescence of the corresponding shRNA subset (**Cs. A. %F.**), i.e. the average % fluorescence (and shRNA #s) for the subset of shRNAs tested with the original long reporters corresponding to each shortened fragment reporter. The last column contains the combined average % fluorescence (**C. A. %F.**), that is, the activities measured form the group 1 accessory gene reporters and the group 2 shortened core gene reporters, tallied to a combined average at the bottom.

**Rep.**	**Length**	**A. %F.**	**Cs. A. %F.**	**Shortened Rep.**	**Length**	**A. %F.**	**C. A. %F.**
Gag	1503	38.38%	-				-
subset #10 - #18		-	39%	Gag-1 (500 - 1150)	650	37%	37%
Pol	3012	41.64%	-				-
subset #19 - #29		-	41%	Pol-1 (1 - 436)	436	21%	21%
subset #30		-	58%	Pol-2 (400 - 850)	450	51%	51%
subset #31 - #34		-	41%	Pol-3 (800 - 1250)	450	20%	20%
subset #35 - #57		-	42%	Pol-4 (2600 - 3025)	425	27%	27%
Tat (x1-2)	258	18.92%	-				19%
Rev (x1-2)	272	12.73%	-				13%
Vpu	243	10.32%	-				10%
Env	2250	52.20%	-				-
subset #77- #91		-	53%	Env-1 (1300 - 1740)	440	18%	18%
**Nef-LTR ***	921	44.36%					46%
**Combined averages :**		**37.2%**	**43.8%**			**25.0%**	**24.9%**

***** *this may be ~ 100 bp shorter than intended due to an internal transcription termination signal within the LTR sequence.*

Ranked selection

We created a scoring method to rank the 96 hairpins for overall performance based on their suppressive activities, non-specific activities and percentage conservations. Each parameter was weighted approximately equally and was awarded a score out of 100 (nb. changing the weighting for any parameter will change the following ranking). Suppressive activities from both assays were tallied as 100 minus the percentage fluorescence value or percentage p24 graphed. Non-specific activity was awarded a top score of 100 for no activity (a normalization factor of 1.0), which was reduced accordingly for increased non-specific activity, e.g. a normalization factor of 1.2 (0.2 fold enhancement) or 0.8 (0.2 fold reduction) was only awarded 80, and so on. The percentage conservations for all strains and the clade B subtypes were also tallied, but only using the p0 position. The top ranking hairpin for each gene is shown in bold. The hairpins sorted in ranked ordered according to score (the last column, **Rk.**). The original values and the scores for each parameter are shown.

		**%F.**		**% p24**		**Non sp.**		**% cons. ALL (p0)**		**% cons. B (p0)**			
**#**	**Target**	**Val.**	**Sc.**	**Val.**	**Sc.**	**Val.**	**Sc.**	**Val.**	**Sc.**	**Val.**	**Sc.**	**C.s.**	**Rk.**
5	**LTR 510-21**	18.7	81.3	1.1	98.9	1.3	70	70	70	100	100	420	**1**
8	LTR 527-21	23.9	76.1	3.9	96.1	1.5	50	93	93	100	100	415	**2**
4	LTR 509-20	35.5	64.5	8.5	91.5	1.2	80	69	69	100	100	405	**3**
83	**Env 1428-21**	7.3	92.7	6.3	93.8	1.4	60	73	73	81	81	401	**4**
58	**Tat (x1) 131-20**	26.3	73.7	16.5	83.5	1.1	90	68	68	84	84	399	**5**
59	Tat (x1) 132-21	26.8	73.2	26.2	73.8	1.0	100	68	68	84	84	399	**6**
81	Env 1425-21	7.2	92.8	6.2	93.9	1.4	60	74	74	77	77	398	**7**
82	Env 1426-21	6.9	93.1	4.3	95.7	1.7	30	79	79	84	84	382	**8**
6	LTR 516-21	14.1	85.9	0.6	99.4	1.9	10	71	71	100	100	366	**9**
11	**Gag 532-21**	40.7	59.3	4.9	95.1	1.4	60	70	70	80	80	364	**10**
79	Env 1424-21	6.5	93.5	10.7	89.3	1.7	30	74	74	77	77	364	**11**
7	LTR 522-20	61.0	39.0	47.4	52.6	1.2	80	92	92	100	100	364	**12**
68	**Vpu 143-20**	6.7	93.3	10.1	89.9	1.7	30	66	66	83	83	362	**13**
80	Env 1425-20	11.3	88.7	21.5	78.6	1.6	40	74	74	77	77	358	**14**
23	**Pol 248-21**	8.8	91.2	0.3	99.7	3.1	0	80	80	87	87	358	**15**
22	Pol 248-20	8.9	91.1	0.5	99.5	2.2	0	80	80	87	87	358	**16**
39	Pol 2726-21	15.4	84.6	4.5	95.5	1.9	10	81	81	86	86	357	**17**
25	Pol 249-21	8.8	91.2	0.5	99.5	2.7	0	79	79	86	86	356	**18**

		%F.		% p24		Non sp.		% cons. ALL (p0)		% cons. B (p0)			
**#**	**Target**	**Val.**	**Sc.**	**Val.**	**Sc.**	**Val.**	**Sc.**	**Val.**	**Sc.**	**Val.**	**Sc.**	**C.s.**	**Rk.**
27	Pol 250-21	9.8	90.2	1.7	98.3	2.8	0	80	80	87	87	356	**19**
62	Tat (x1) 147-20	17.0	83.0	5.7	94.3	1.5	50	60	60	68	68	355	**20**
26	Pol 250-20	9.8	90.2	2.0	98.0	2.5	0	80	80	87	87	355	**21**
24	Pol 249-20	9.2	90.8	1.0	99.0	3.3	0	79	79	86	86	355	**22**
37	Pol 2671-21	9.9	90.1	2.7	97.3	1.8	20	73	73	74	74	354	**23**
21	Pol 247-21	10.0	90.0	2.7	97.3	3.0	0	80	80	87	87	354	**24**
20	Pol 246-21	8.5	91.5	0.5	99.5	4.2	0	80	80	81	81	352	**25**
38	Pol 2724-20	13.8	86.2	4.3	95.7	1.7	30	71	71	67	67	350	**26**
29	Pol 404-20	23.8	76.2	38.7	61.3	0.8	80	67	67	65	65	350	**27**
64	Vpu 139-21	8.9	91.1	15.0	85.0	1.7	30	64	64	77	77	347	**28**
44	Pol 2878-21	8.0	92.0	1.5	98.5	2.6	0	74	74	80	80	345	**29**
43	Pol 2878-20	7.6	92.4	2.5	97.5	2.6	0	74	74	80	80	344	**30**
45	Pol 2879-20	8.1	91.9	1.2	98.8	2.1	0	74	74	79	79	344	**31**
57	**Vif 37-20**	9.5	90.5	4.9	95.1	2.7	0	71	71	87	87	344	**32**
52	Vif 10-21	11.0	89.0	9.7	90.3	7.2	0	71	71	92	92	342	**33**
42	Pol 2874-21	20.3	79.7	15.8	84.2	3.1	0	85	85	93	93	342	**34**
49	Pol 2882-21	8.8	91.2	3.5	96.5	3.4	0	74	74	80	80	342	**35**
35	Pol 2668-21	17.1	82.9	4.8	95.2	2.6	0	78	78	84	84	340	**36**
53	Vif 11-21	15.7	84.3	9.4	90.6	3.4	0	71	71	92	92	338	**37**
48	Pol 2881-21	12.1	87.9	5.7	94.3	3.1	0	74	74	80	80	336	**38**
36	Pol 2670-21	9.5	90.5	2.0	98.0	2.0	0	73	73	74	74	336	**39**
71	Vpu 145-20	6.8	93.2	7.7	92.3	2.6	0	66	66	83	83	335	**40**
13	Gag 533-21	31.1	68.9	5.2	94.8	1.8	20	70	70	80	80	334	**41**
72	Vpu 146-20	7.3	92.7	11.7	88.3	2.1	0	67	67	84	84	332	**42**
77	Env 1423-20	6.1	93.9	10.2	89.9	3.0	0	72	72	76	76	332	**43**
46	Pol 2879-21	95.8	4.2	15.7	84.3	1.1	90	74	74	79	79	332	**44**
56	Vif 15-21	22.6	77.4	6.4	93.6	2.8	0	70	70	90	90	331	**45**
55	Vif 14-21	13.9	86.1	15.6	84.4	4.5	0	70	70	90	90	331	**46**
17	Gag 578-20	35.3	64.7	26.8	73.2	1.5	50	63	63	79	79	330	**47**
60	Tat (x1) 140-21	14.4	85.6	5.7	94.3	2.6	0	73	73	77	77	330	**48**
78	Env 1423-21	8.3	91.7	11.5	88.5	2.6	0	72	72	76	76	328	**49**
70	Vpu 144-21	5.9	94.1	14.0	86.0	2.3	0	65	65	83	83	328	**50**
47	Pol 2880-20	29.2	70.8	16.0	84.0	1.8	20	74	74	79	79	328	**51**

		%F.		% p24		Non sp.		% cons. ALL (p0)		% cons. B (p0)			
**#**	**Target**	**Val.**	**Sc.**	**Val.**	**Sc.**	**Val.**	**Sc.**	**Val.**	**Sc.**	**Val.**	**Sc.**	**C.s.**	**Rk.**
51	Vif 9-21	16.6	83.4	18.9	81.1	6.7	0	71	71	92	92	328	**52**
86	Env 1432-21	7.3	92.7	32.0	68.0	1.8	20	65	65	81	81	327	**53**
19	Pol 242-20	8.0	92.0	0.5	99.5	2.0	0	58	58	77	77	327	**54**
14	Gag 534-20	40.1	59.9	14.7	85.3	1.7	30	69	69	80	80	324	**55**
30	Pol 529-21	51.2	48.8	29.2	70.8	1.5	50	68	68	84	84	322	**56**
69	Vpu 144-20	6.8	93.2	21.4	78.6	2.5	0	65	65	83	83	320	**57**
32	Pol 925-20	14.5	85.5	2.7	97.3	3.6	0	60	60	77	77	320	**58**
12	Gag 533-20	27.1	72.9	4.1	95.9	2.1	0	70	70	80	80	319	**59**
10	Gag 532-20	55.4	44.6	46.0	54.1	1.3	70	70	70	80	80	319	**60**
9	LTR 553-20	61.5	38.5	33.0	67.0	1.3	70	51	51	92	92	319	**61**
96	**Nef 351-20**	38.5	61.5	36.3	63.7	1.2	80	49	49	62	62	316	**62**
61	Tat (x1) 143-21	14.0	86.0	2.3	97.7	2.1	0	62	62	70	70	316	**63**
54	Vif 12-20	12.8	87.2	30.8	69.2	5.1	0	70	70	89	89	315	**64**
89	Env 1434-20	19.9	80.1	78.2	21.9	1.3	70	63	63	79	79	314	**65**
67	Vpu 141-20	6.5	93.5	8.5	91.5	2.4	0	56	56	72	72	313	**66**
18	Gag 698-20	20.9	79.1	12.2	87.9	5.0	0	70	70	76	76	313	**67**
15	Gag 534-21	44.0	56.0	33.7	66.4	1.6	40	69	69	80	80	311	**68**
33	Pol 926-20	20.3	79.7	8.5	91.5	2.2	0	60	60	77	77	308	**69**
73	Vpu 147-21	6.2	93.8	5.8	94.2	2.7	0	53	53	63	63	304	**70**
1	LTR 63-20	56.4	43.6	48.8	51.2	1.0	100	33	33	76	76	304	**71**
90	Env 1436-20	42.3	57.7	88.1	12.0	1.1	90	64	64	80	80	304	**72**
63	**Rev (x1) 9-20**	15.0	85.0	11.6	88.4	2.9	0	60	60	68	68	301	**73**
74	Vpu 148-21	6.0	94.0	8.2	91.8	2.7	0	53	53	62	62	301	**74**
66	Vpu 140-21	24.6	75.4	67.4	32.6	1.5	50	64	64	78	78	300	**75**
16	Gag 535-20	33.9	66.1	51.4	48.6	1.7	30	71	71	82	82	298	**76**
41	Pol 2873-21	39.5	60.5	45.8	54.2	3.4	0	88	88	95	95	298	**77**
75	Vpu 149-20	7.8	92.2	10.3	89.7	2.2	0	53	53	62	62	297	**78**
87	Env 1433-20	18.2	81.8	89.9	10.1	1.5	50	65	65	81	81	288	**79**
31	Pol 921-20	27.2	72.8	19.0	81.0	4.0	0	58	58	76	76	288	**80**
88	Env 1433-21	18.9	81.1	72.6	0.0	1.4	60	65	65	81	81	287	**81**
3	LTR 507-20	78.7	21.3	102	-1.8	1.0	100	69	69	98	98	287	**82**
91	Env 1437-20	63.0	37.0	99.9	0.0	1.0	100	66	66	83	83	286	**83**
34	Pol 1161-20	19.6	80.4	6.5	93.5	2.3	0	32	32	75	75	281	**84**

		%F.		% p24		Non sp.		% cons. ALL (p0)		% cons. B (p0)			
**#**	**Target**	**Val.**	**Sc.**	**Val.**	**Sc.**	**Val.**	**Sc.**	**Val.**	**Sc.**	**Val.**	**Sc.**	**C.s.**	**Rk.**
76	Env 628-20	47.7	52.3	68.3	31.8	1.5	50	61	61	85	85	280	**85**
95	Nef 350-20	27.6	72.4	28.9	0.0	0.9	90	51	51	64	64	277	**86**
92	Env 2308-20	18.8	81.2	35.6	64.4	2.2	0	43	43	74	74	263	**87**
2	LTR 506-20	72.0	28.0	85.1	14.9	1.6	40	67	67	97	97	247	**88**
85	Env 1432-20	12.0	88.0	40.5	0.0	2.0	0	65	65	81	81	234	**89**
28	Pol 251-20	125	-25	88.7	11.3	0.8	80	80	80	87	87	234	**90**
40	Pol 2750-20	122	-22	0.8	99.2	1.9	10	65	65	75	75	227	**91**
93	Rev (x2) 158-20	8.0	0.0	2.5	97.5	2.1	0	48	48	76	76	222	**92**
84	Env 1431-21	28.4	71.6	118	-18	1.8	20	65	65	81	81	220	**93**
65	Vpu 140-20	30.3	69.7	156	-56	1.4	60	64	64	78	78	215	**94**
94	Rev (x2) 159-20	11.4	0.0	10.1	89.9	3.4	0	48	48	75	75	213	**95**
50	Pol 2885-20	103	-2.6	212	-112	0.8	80	64	64	79	79	108	**96**

Additional methods

Oligonucleotide templates for Phi-29 extension

The coding region for each hairpin was contained within a single oligonucleotide, organized in the following general format: 5’-GCGCGGATCC | core(20 - 21) | NCTCGAGN | core(20 - 21) | [G/A/C] | TTTTTTGGAAGCTT-3’. Each oligonucleotide encoded a digestion ‘seat’ (for the RE to better bind the oligonucleotide), a 5’ RE recognition site (*Bam* HI), the shRNA sense stem region, loop sequence, anti-sense stem, a termination spacer (if required), a pol III termination signal consisting of a run of at least 4 ‘T’s, a 3’ RE recognition site (*Hind* III) and a 3’ digestion seat. The termination spacer was included only if the last base of the anti-sense stem was ‘T’, so as to prevent premature termination via an early run of ‘T’s. A short primer was designed to bind at the 3’ end of the template oligo and introduced the 3’ seat (5’-CGCGAAGCTTCCAAAAAA-3’).

Oligos were ordered at the minimal synthesis and purification scales (0.05 μM and desalt, Sigma-Genosys) and were re-suspended in water (1 - 10 μg / μl). Twenty picomoles of each oligo was used in the extension reaction (1x reaction buffer, 2x BSA, 20 μM dNTPs (1μl of a 10 mM stock), 10 units of Phi29 (New England Biolabs) and water to 20 μl), which was incubated at 30 °C for ~10 min., then 65 °C for 10 min. (to disable the polymerase). The extension product was digested (*Bam* HI plus *Hind* III), purified using the Nucleotide Removal kit (Qiagen), ligated to the expression plasmid and used to transform electrocompetent GT116 E.*coli*. Positive clones were confirmed by automated sequencing using our loop digestion method (see below). The following table lists the **93** oligonucleotide template sequences ordered as Phi-29 extension templates.

Hairpin #	Target (NL4-3)	Oligonucleotide template (5’ to 3’)
1	LTR 63-20	GCGCGGATCCCAAGGCTACTTCCCTGATTGCCTCGAGCCAATCAGG GAAGTAGCCTTGTTTTTTGGAAGCTT
2	LTR 506-20	GCGCGGATCCGGAACCCACTGCTTAAGCCTGCTCGAGGAGGCTTAA GCAGTGGGTTCCTTTTTTGGAAGCTT
3	LTR 507-20	GCGCGGATCCGAACCCACTGCTTAAGCCTCTCTCGAGTGAGGCTTAA GCAGTGGGTTCTTTTTTGGAAGCTT
4	LTR 509-20	GCGCGGATCCACCCACTGCTTAAGCCTCAAACTCGAGATTGAGGCTT AAGCAGTGGGTTTTTTTGGAAGCTT
5	LTR 510-21	GCGCGGATCCCCCACTGCTTAAGCCTCAATAACTCGAGATATTGAGG CTTAAGCAGTGGGTTTTTTGGAAGCTT
6	LTR 516-21	Overlapping oligonucleotide pair used instead, see below
7	LTR 522-20	Overlapping oligonucleotide pair used instead, see below

Hairpin #	Target (NL4-3)	Oligonucleotide template (5' to 3')
8	LTR 527-21	Overlapping oligonucleotide pair used instead, see below
9	LTR 553-20	GCGCGGATCCGTAGTGTGTGCCCGTCTGTTCCTCGAGCAACAGACG GGCACACACTACTTTTTTGGAAGCTT
10	Gag 532-20	GCGCGGATCCGGAGCCACCCCACAAGATTTTCTCGAGTAAATCTTGT GGGGTGGCTCCTTTTTTGGAAGCTT
11	Gag 532-21	GCGCGGATCCGGAGCCACCCCACAAGATTTAACTCGAGATAAATCTT GTGGGGTGGCTCCTTTTTTGGAAGCTT
12	Gag 533-20	GCGCGGATCCGAGCCACCCCACAAGATTTATCTCGAGTTAAATCTTGT GGGGTGGCTCTTTTTTGGAAGCTT
13	Gag 533-21	GCGCGGATCCGAGCCACCCCACAAGATTTAAACTCGAGATTAAATCTT GTGGGGTGGCTCTTTTTTGGAAGCTT
14	Gag 534-20	GCGCGGATCCAGCCACCCCACAAGATTTAATCTCGAGTTTAAATCTTG TGGGGTGGCTCTTTTTTGGAAGCTT
15	Gag 534-21	GCGCGGATCCAGCCACCCCACAAGATTTAAAACTCGAGATTTAAATCT TGTGGGGTGGCTCTTTTTTGGAAGCTT
16	Gag 535-20	GCGCGGATCCGCCACCCCACAAGATTTAAAACTCGAGATTTAAATCTT GTGGGGTGGCTTTTTTGGAAGCTT
17	Gag 578-20	GCGCGGATCCGACATCAAGCAGCCATGCAATCTCGAGTTTGCATGGC TGCTTGATGTCTTTTTTGGAAGCTT
18	Gag 698-20	GCGCGGATCCGAAGTGACATAGCAGGAACTTCTCGAGTAGTTCCTGC TATGTCACTTCTTTTTTGGAAGCTT
19	Pol 242-20	GCGCGGATCCATACAGGAGCAGATGATACACCTCGAGCTGTATCATCT GCTCCTGTATCTTTTTTGGAAGCTT
20	Pol 246-21	GCGCGGATCCAGGAGCAGATGATACAGTATTACTCGAGAAATACTGTA TCATCTGCTCCTGTTTTTTGGAAGCTT
21	Pol 247-21	GCGCGGATCCGGAGCAGATGATACAGTATTAACTCGAGATAATACTGTA TCATCTGCTCCTTTTTTGGAAGCTT
22	Pol 248-20	GCGCGGATCCGAGCAGATGATACAGTATTACCTCGAGCTAATACTGTAT CATCTGCTCTTTTTTGGAAGCTT
23	Pol 248-21	GCGCGGATCCGAGCAGATGATACAGTATTAGACTCGAGACTAATACTG TATCATCTGCTCTTTTTTGGAAGCTT
24	Pol 249-20	GCGCGGATCCAGCAGATGATACAGTATTAGTCTCGAGTCTAATACTGTA TCATCTGCTCTTTTTTGGAAGCTT
25	Pol 249-21	GCGCGGATCCAGCAGATGATACAGTATTAGAACTCGAGATCTAATACT GTATCATCTGCTCTTTTTTGGAAGCTT
26	Pol 250-20	GCGCGGATCCGCAGATGATACAGTATTAGATCTCGAGTTCTAATACTGT ATCATCTGCTTTTTTGGAAGCTT
27	Pol 250-21	GCGCGGATCCGCAGATGATACAGTATTAGAAACTCGAGATTCTAATACT GTATCATCTGCTTTTTTGGAAGCTT
28	Pol 251-20	GCGCGGATCCCAGATGATACAGTATTAGAACCTCGAGCTTCTAATACT GTATCATCTGTTTTTTGGAAGCTT

Hairpin #	Target (NL4-3)	Oligonucleotide template (5' to 3')
29	Pol 404-20	GCGCGGATCCCTACACCTGTCAACATAATTCCTCGAGCAATTATGTTG ACAGGTGTAGTTTTTTGGAAGCTT
30	Pol 529-21	GCGCGGATCCAAACAATGGCCATTGACAGAAACTCGAGATTCTGTCA ATGGCCATTGTTTATTTTTTGGAAGCTT
31	Pol 921-20	GCGCGGATCCATGGAAAGGATCACCAGCAAACTCGAGATTGCTGGTG ATCCTTTCCATCTTTTTTGGAAGCTT
32	Pol 925-20	GCGCGGATCCAAAGGATCACCAGCAATATTGCTCGAGGAATATTGCTG GTGATCCTTTCTTTTTTGGAAGCTT
33	Pol 926-20	GCGCGGATCCAAGGATCACCAGCAATATTCGCTCGAGGGAATATTGCT GGTGATCCTTTTTTTTGGAAGCTT
34	Pol 1161-20	GCGCGGATCCTGAACTCCATCCTGATAAATCCTCGAGCATTTATCAGG ATGGAGTTCATTTTTTGGAAGCTT
35	Pol 2668-21	GCGCGGATCCGCAGTACAAATGGCAGTATTCACTCGAGAGAATACTG CCATTTGTACTGCTTTTTTGGAAGCTT
36	Pol 2670-21	GCGCGGATCCAGTACAAATGGCAGTATTCATACTCGAGAATGAATACT GCCATTTGTACTGTTTTTTGGAAGCTT
37	Pol 2671-21	GCGCGGATCCGTACAAATGGCAGTATTCATCACTCGAGAGATGAATAC TGCCATTTGTACTTTTTTGGAAGCTT
38	Pol 2724-20	GCGCGGATCCGTACAGTGCAGGGGAAAGAAACTCGAGATTCTTTCCC CTGCACTGTACTTTTTTGGAAGCTT
39	Pol 2726-21	GCGCGGATCCACAGTGCAGGGGAAAGAATAGACTCGAGACTATTCTT TCCCCTGCACTGTATTTTTTGGAAGCTT
40	Pol 2750-20	GCGCGGATCCACATAATAGCAACAGACATAGCTCGAGGTATGTCTGTT GCTATTATGTCTTTTTTGGAAGCTT
41	Pol 2873-21	GCGCGGATCCGGAAAGGTGAAGGGGCAGTAGACTCGAGACTACTGC CCCTTCACCTTTCCTTTTTTGGAAGCTT
42	Pol 2874-21	GCGCGGATCCGAAAGGTGAAGGGGCAGTAGTACTCGAGAACTACTG CCCCTTCACCTTTCTTTTTTGGAAGCTT
43	Pol 2878-20	GCGCGGATCCGGTGAAGGGGCAGTAGTAATTCTCGAGTATTACTACTG CCCCTTCACCTTTTTTGGAAGCTT
44	Pol 2878-21	GCGCGGATCCGGTGAAGGGGCAGTAGTAATAACTCGAGATATTACTAC TGCCCCTTCACCTTTTTTGGAAGCTT
45	Pol 2879-20	GCGCGGATCCGTGAAGGGGCAGTAGTAATAGCTCGAGGTATTACTACT GCCCCTTCACTTTTTTGGAAGCTT
46	Pol 2879-21	GCGCGGATCCGTGAAGGGGCAGTAGTAATACACTCGAGAGTATTACTA CTGCCCCTTCACTTTTTTGGAAGCTT
47	Pol 2880-20	GCGCGGATCCTGAAGGGGCAGTAGTAATACTCTCGAGTGTATTACTAC TGCCCCTTCATTTTTTGGAAGCTT
48	Pol 2881-21	GCGCGGATCCGAAGGGGCAGTAGTAATACAAACTCGAGATTGTATTAC TACTGCCCCTTCTTTTTTGGAAGCTT
49	Pol 2882-21	GCGCGGATCCAAGGGGCAGTAGTAATACAAGACTCGAGACTTGTATTA CTACTGCCCCTTCTTTTTTGGAAGCTT

Hairpin #	Target (NL4-3)	Oligonucleotide template (5' to 3')
50	Pol 2885-20	GCGCGGATCCGGGCAGTAGTAATACAAGATTCTCGAGTATCTTGTATTA CTACTGCCCTTTTTTGGAAGCTT
51	Vif 9-21	GCGCGGATCCCAGATGGCAGGTGATGATTGTACTCGAGAACAATCAT CACCTGCCATCTGTTTTTTGGAAGCTT
52	Vif 10-21	GCGCGGATCCAGATGGCAGGTGATGATTGTGACTCGAGACACAATCA TCACCTGCCATCTGTTTTTTGGAAGCTT
53	Vif 11-21	GCGCGGATCCGATGGCAGGTGATGATTGTGTACTCGAGAACACAATC ATCACCTGCCATCTTTTTTGGAAGCTT
54	Vif 12-20	GCGCGGATCCATGGCAGGTGATGATTGTGTCCTCGAGCACACAATCA TCACCTGCCATCTTTTTTGGAAGCTT
55	Vif 14-21	GCGCGGATCCGGCAGGTGATGATTGTGTGGCACTCGAGAGCCACAC AATCATCACCTGCCTTTTTTGGAAGCTT
56	Vif 15-21	GCGCGGATCCGCAGGTGATGATTGTGTGGCAACTCGAGATGCCACA CAATCATCACCTGCTTTTTTGGAAGCTT
57	Vif 37-20	GCGCGGATCCGTAGACAGGATGAGGATTAAGCTCGAGGTTAATCCTC ATCCTGTCTACTTTTTTGGAAGCTT
58	Tat (exon 1) 131-20	GCGCGGATCCGCATCTCCTATGGCAGGAAGTCTCGAGTCTTCCTGCC ATAGGAGATGCTTTTTTGGAAGCTT
59	Tat (exon 1) 132-21	GCGCGGATCCCATCTCCTATGGCAGGAAGAAACTCGAGATTCTTCCT GCCATAGGAGATGTTTTTTGGAAGCTT
60	Tat (exon 1) 140-21	GCGCGGATCCATGGCAGGAAGAAGCGGAGACACTCGAGAGTCTCC GCTTCTTCCTGCCATATTTTTTGGAAGCTT
61	Tat (exon 1) 143-21	GCGCGGATCCGCAGGAAGAAGCGGAGACAGCACTCGAGAGCTGTC TCCGCTTCTTCCTGCTTTTTTGGAAGCTT
62	Tat (exon 1) 147-20	GCGCGGATCCGAAGAAGCGGAGACAGCGACCCTCGAGCGTCGCTG TCTCCGCTTCTTCTTTTTTGGAAGCTT
63	Rev (exon 1) 9-20	GCGCGGATCCAAGAAGCGGAGACAGCGACGTCTCGAGTCGTCGCT GTCTCCGCTTCTTCTTTTTTGGAAGCTT
64	Vpu 139-21	GCGCGGATCCGAAAGAGCAGAAGACAGTGGCACTCGAGAGCCACT GTCTTCTGCTCTTTCTTTTTTGGAAGCTT
65	Vpu 140-20	GCGCGGATCCAAAGAGCAGAAGACAGTGGCTCTCGAGTGCCACTGT CTTCTGCTCTTTCTTTTTTGGAAGCTT
66	Vpu 140-21	GCGCGGATCCAAAGAGCAGAAGACAGTGGCAACTCGAGATGCCACT GTCTTCTGCTCTTTCTTTTTTGGAAGCTT
67	Vpu 141-20	GCGCGGATCCAAGAGCAGAAGACAGTGGCATCTCGAGTTGCCACTG TCTTCTGCTCTTTTTTTTGGAAGCTT
68	Vpu 143-20	GCGCGGATCCGAGCAGAAGACAGTGGCAATCCTCGAGCATTGCCAC TGTCTTCTGCTCTTTTTTGGAAGCTT
69	Vpu 144-20	GCGCGGATCCAGCAGAAGACAGTGGCAATGTCTCGAGTCATTGCCA CTGTCTTCTGCTCTTTTTTGGAAGCTT
70	Vpu 144-21	GCGCGGATCCAGCAGAAGACAGTGGCAATGAACTCGAGATCATTGC CACTGTCTTCTGCTCTTTTTTGGAAGCTT

Hairpin #	Target (NL4-3)	Oligonucleotide template (5' to 3')
71	Vpu 145-20	GCGCGGATCCGCAGAAGACAGTGGCAATGACCTCGAGCTCATTGCC ACTGTCTTCTGCTTTTTTGGAAGCTT
72	Vpu 146-20	GCGCGGATCCCAGAAGACAGTGGCAATGAGTCTCGAGTCTCATTGC CACTGTCTTCTGTTTTTTGGAAGCTT
73	Vpu 147-21	GCGCGGATCCAGAAGACAGTGGCAATGAGAGACTCGAGACTCTCATT GCCACTGTCTTCTGTTTTTTGGAAGCTT
74	Vpu 148-21	GCGCGGATCCGAAGACAGTGGCAATGAGAGTACTCGAGAACTCTCAT TGCCACTGTCTTCTTTTTTGGAAGCTT
75	Vpu 149-20	GCGCGGATCCAAGACAGTGGCAATGAGAGTCCTCGAGCACTCTCATT GCCACTGTCTTCTTTTTTGGAAGCTT
76	Env 628-20	GCGCGGATCCCCAATTCCCATACATTATTGACTCGAGACAATAATGTAT GGGAATTGGTTTTTTGGAAGCTT
77	Env 1423-20	GCGCGGATCCGACAATTGGAGAAGTGAATTTCTCGAGTAATTCACTTC TCCAATTGTCTTTTTTGGAAGCTT
78	Env 1423-21	GCGCGGATCCGACAATTGGAGAAGTGAATTAACTCGAGATAATTCACT TCTCCAATTGTCTTTTTTGGAAGCTT
79	Env 1424-21	GCGCGGATCCACAATTGGAGAAGTGAATTATACTCGAGAATAATTCACT TCTCCAATTGTCTTTTTTGGAAGCTT
80	Env 1425-20	GCGCGGATCCCAATTGGAGAAGTGAATTATTCTCGAGTATAATTCACTT CTCCAATTGTTTTTTGGAAGCTT
81	Env 1425-21	GCGCGGATCCCAATTGGAGAAGTGAATTATAACTCGAGATATAATTCAC TTCTCCAATTGTTTTTTGGAAGCTT
82	Env 1426-21	GCGCGGATCCAATTGGAGAAGTGAATTATATACTCGAGAATATAATTCA CTTCTCCAATTGTTTTTTGGAAGCTT
83	Env 1428-21	GCGCGGATCCTTGGAGAAGTGAATTATATAAACTCGAGATTATATAATTC ACTTCTCCAATTTTTTGGAAGCTT
84	Env 1431-21	GCGCGGATCCGAGAAGTGAATTATATAAATAACTCGAGATATTTATATAAT TCACTTCTCTTTTTTGGAAGCTT
85	Env 1432-20	GCGCGGATCCAGAAGTGAATTATATAAATAACTCGAGATATTTATATAATT CACTTCTCTTTTTTGGAAGCTT
86	Env 1432-21	GCGCGGATCCAGAAGTGAATTATATAAATATACTCGAGAATATTTATATAA TTCACTTCTCTTTTTTGGAAGCTT
87	Env 1433-20	GCGCGGATCCGAAGTGAATTATATAAATATTCTCGAGTATATTTATATAATT CACTTCTTTTTTGGAAGCTT
88	Env 1433-21	GCGCGGATCCGAAGTGAATTATATAAATATAACTCGAGATATATTTATATA ATTCACTTCTTTTTTGGAAGCTT
89	Env 1434-20	GCGCGGATCCAAGTGAATTATATAAATATATCTCGAGTTATATTTATATAAT TCACTTCTTTTTTGGAAGCTT
90	Env 1436-20	GCGCGGATCCGTGAATTATATAAATATAAACCTCGAGCTTTATATTTATAT AATTCACTTTTTTGGAAGCTT
91	Env 1437-20	GCGCGGATCCTGAATTATATAAATATAAAGACTCGAGACTTTATATTTATA TAATTCATTTTTTGGAAGCTT

Hairpin #	Target (NL4-3)	Oligonucleotide template (5' to 3')
92	Env 2308-20	GCGCGGATCCAGAGACTTACTCTTGATTGTTCTCGAGTACAATCAAGA GTAAGTCTCTCTTTTTTGGAAGCTT
93	Rev (exon 2) 158-20	GCGCGGATCCTGAGAGACTTACTCTTGATTCCTCGAGCAATCAAGAG TAAGTCTCTCATTTTTTGGAAGCTT
94	Rev (exon 2) 159-20	GCGCGGATCCGAGAGACTTACTCTTGATTGACTCGAGACAATCAAGA GTAAGTCTCTCTTTTTTGGAAGCTT
95	Nef 350-20	GCGCGGATCCCACAAGGCTACTTCCCTGATACTCGAGAATCAGGGAA GTAGCCTTGTGTTTTTTGGAAGCTT
96	Nef 351-20	GCGCGGATCCACAAGGCTACTTCCCTGATTCCTCGAGCAATCAGGGA AGTAGCCTTGTGTTTTTTGGAAGCTT

n.b. The target positions are the co-ordinates within each gene of NL4-3.

Oligonucleotide pairs for making synthetic inserts with offset ends

Three hairpin templates were created from standard complementary oligonucleotide pairs with offset ends as each hairpin core sequence contained an internal *Hind* III site making it incompatible with the Phi-29 extension method. These templates were made as synthetic duplexes with overhanging ends identical to those created by restriction enzyme (RE) digestion (*Bam* HI at the 5' and *Hind* III at the 3'). The coding region for each hairpin was contained within a single oligonucleotide (upper oligo: 5'-GATCC | core(20 - 21) | NCTCGAGN | core(20 - 21) | [G/A/C] | TTTTTTGGA-3') and its complementary equivalent (lower oligo: 5'-AGCTTCCAAAAAA | [G/A/C] | core(20 - 21) | NCTCGAGN | core(20 - 21) | G-3'). Both oligos were synthesized and re-suspended as previously described. 1 µl from each was added to 98 µl of annealing solution (10 mM Tris pH 8.0, 50 mM NaCl, 1 mM EDTA), heated to 100 °C for 5 minutes, slowly equilibrated to room temperature and diluted up to 10,000 fold for ligation. The inserts and plasmids were ligated, and used to transform electrocompetent GT116 E.*coli* (Invivogen). The following table lists the **3** hairpin templates ordered as complementary oligonucleotide pairs.

Hairpin #	Target	Oligonucleotide template (5' to 3')
6 (lower)	LTR 516-21	AGCTTTCCAAAAAAGCTTAAGCCTCAATAAAGCTTTCTCGAGTAAGCTT TATTGAGGCTTAAGCG
6 (upper)	"	GATCCGCTTAAGCCTCAATAAAGCTTACTCGAGAAAGCTTTATTGAGG CTTAAGCTTTTTTGGA
7 (lower)	LTR 522-20	AGCTTTCCAAAAAAGCCTCAATAAAGCTTGCCTTGCTCGAGGAAGGC AAGCTTTATTGAGGCG
7 (upper)	"	GATCCGCCTCAATAAAGCTTGCCTTCCTCGAGCAAGGCAAGCTTTATT GAGGCTTTTTTGGA
8 (lower)	LTR 527-21	AGCTTTCCAAAAAACAATAAAGCTTGCCTTGAGTGCTCTCGAGTGCAC TCAAGGCAAGCTTTATTG

Hairpin #	Target	Oligonucleotide template (5' to 3')
8 (upper)	"	GATCCAATAAAGCTTGCCTTGAGTGCACTCGAGAGCACTCAAGGCAAGCTTTATTGTTTTTTGGA

Oligonucleotide templates for the off-target control shRNA

The off-target hairpin plasmid was taken from previous work, but was originally constructed using standard complementary oligonucleotide pairs as described above. The following table lists the corresponding complementary oligonucleotide pairs, with the stem region indicated in light grey.

Hairpin #	Target	Oligonucleotide template (5' to 3')
off target (upper)	none	GATCCAAGACAGTCCAACACACGCCACCTGTCTCTCTCGAGTGAGACAGGTGGCGTGTGTTGGACTGTCTTTTTTTTGGA
off target (lower)	"	AGCTTTCCAAAAAAAAGACAGTCCAACACACGCCACCTGTCTCACTCGAGAGAGACAGGTGGCGTGTGTTGGACTGTCTTG

shRNA plasmid sequence confirmation by loop digestion

Each construct was digested and sequenced in two reactions, one containing the forward primer, the other containing the reverse. The primers bound to the expression plasmid backbone approximately 100 bases away from the region encoding the base of the hairpin stem. Each reaction contained: 1x RE buffer (NEB-2, New England Biolabs), 1x BSA, ~ 500 ng of template plasmid, 10 pmol of sequencing primer, ~ 10 units XhoI and water to a total volume of 16 µl, and was incubated at 37 °C for 30 - 60 min. prior to shipping, without purification, to an automated sequencing facility (Australian Genome Research Facility, AGRF).

The gene sequences

The HIV-1 hairpins used in this project were designed to target the common laboratory strain, NL4-3 [Genbank:**AF324493**]. The gene sequences from this strain were as follows:

LTR

tggaagggctaattcactcccaaagaagacaagatatccttgatctgtggatctaccacacacaaggctacttccctgattgg
cagaactacacaccagggccaggggtcagatatccactgacctttggatggtgctacaagctagtaccagttgagccagat
aaggtagaagaggccaataaaggagagaacaccagcttgttacaccctgtgagcctgcatggaatggatgaccctgagag
agaagtgttagagtggaggtttgacagccgcctagcatttcatcacgtggcccgagagctgcatccggagtacttcaagaac
tgctgacatcgagcttgctacaagggactttccgctggggactttccagggaggcgtggcctgggcgggactggggagtgg
cgagccctcagatgctgcatataagcagctgctttttgcctgtactgggtctctctggttagaccagatctgagcctgggagctc
tctggctaactagggaacccactgcttaagcctcaataaagcttgccttgagtgcttcaagtagtgtgtgcccgtctgttgtgtga
ctctggtaactagagatccctcagacccttttagtcagtgtggaaaatctctagca

Gag

atgggtgcgagagcgtcggtattaagcgggggagaattagataaatgggaaaaaattcggttaaggccagggggaaagaa
acaatataaactaaaacatatagtatgggcaagcagggagctagaacgattcgcagttaatcctggccttttagagacatcag
aaggctgtagacaaatactgggacagctacaaccatcccttcagacaggatcagaagaacttagatcattatataatacaata
gcagtcctctattgtgtgcatcaaaggatagatgtaaaagacaccaaggaagccttagataagatagaggaagagcaaaac
aaaagtaagaaaaaggcacagcaagcagcagctgacacaggaaacaacagccaggtcagccaaaattaccctatagtg
cagaacctccaggggcaaatggtacatcaggccatatcacctagaactttaaatgcatgggtaaaagtagtagaagagaag
gctttcagcccagaagtaatacccatgttttcagcattatcagaaggagccaccccacaagatttaaataccatgctaaacac
agtgggggacatcaagcagccatgcaaatgttaaaagagaccatcaatgaggaagctgcagaatgggatagattgcatc
cagtgcatgcagggcctattgcaccaggccagatgagagaaccaaggggaagtgacatagcaggaactactagtaccctt
caggaacaaataggatggatgacacataatccacctatcccagtaggagaaatctataaaagatggataatcctgggattaa
ataaaatagtaagaatgtatagccctaccagcattctggacataagacaaggaccaaaggaaccctttagagactatgtaga
ccgattctataaaactctaagagccgagcaagcttcacaagaggtaaaaaattggatgacagaaaccttgttggtccaaaat
gcgaacccagattgtaagactattttaaaagcattgggaccaggagcgacactagaagaaatgatgacagcatgtcaggga
gtggggggacccggccataaagcaagagttttggctgaagcaatgagccaagtaacaaatccagctaccataatgataca
gaaaggcaattttaggaaccaaagaaagactgttaagtgtttcaattgtggcaaagaagggcacatagccaaaaattgcagg
gcccctaggaaaaagggctgttggaaatgtggaaaggaaggacaccaaatgaaagattgtactgagagacaggctaattttt
tagggaagatctggccttcccacaagggaaggccagggaattttcttcagagcagaccagagccaacagccccaccaga
agagagcttcaggtttggggaagagacaacaactccctctcagaagcaggagccgatagacaaggaactgtatccctttagc
ttccctcagatcactctttggcagcgacccctcgtcacaataa

Pol

tttttagggaagatctggccttcccacaagggaaggccagggaattttcttcagagcagaccagagccaacagccccacc
agaagagagcttcaggtttggggaagagacaacaactccctctcagaagcaggagccgatagacaaggaactgtatccttt

agcttccctcagatcactctttggcagcgacccctcgtcacaataaagataggggggcaattaaaggaagctctattagatac
aggagcagatgatacagtattagaagaaatgaatttgccaggaagatggaaaccaaaaatgatagggggaattggaggtttt
atcaaagtaagacagtatgatcagatactcatagaaatctgcggacataaagctataggtacagtattagtaggacctacacc
tgtcaacataattggaagaaatctgttgactcagattggctgcactttaaattttcccattagtcctattgagactgtaccagtaaa
attaaagccaggaatggatggcccaaaagttaaacaatggccattgacagaagaaaaaataaaagcattagtagaaatttgt
acagaaatggaaaaggaaggaaaaatttcaaaaattgggcctgaaaatccatacaatactccagtatttgccataaagaaaa
aagacagtactaaatggagaaaattagtagatttcagagaacttaataagagaactcaagatttctgggaagttcaattaggaa
taccacatcctgcagggttaaaacagaaaaaatcagtaacagtactggatgtgggcgatgcatatttttcagttcccttagataa
agacttcaggaagtatactgcatttaccatacctagtataaacaatgagacaccagggattagatatcagtacaatgtgcttcc
acagggatggaaaggatcaccagcaatattccagtgtagcatgacaaaaatcttagagccttttagaaaacaaaatccagac
atagtcatctatcaatacatggatgatttgtatgtaggatctgacttagaaatagggcagcatagaacaaaaatagaggaactg
agacaacatctgttgaggtggggatttaccacaccagacaaaaaacatcagaaagaacctccattcctttggatgggttatga
actccatcctgataaatggacagtacagcctatagtgctgccagaaaaggacagctggactgtcaatgacatacagaaatta
gtgggaaaattgaattgggcaagtcagatttatgcagggattaaagtaaggcaattatgtaaacttcttaggggaaccaaagc
actaacagaagtagtaccactaacagaagaagcagagctagaactggcagaaaacagggagattctaaaagaaccggta
catggagtgtattatgacccatcaaaagacttaatagcagaaatacagaagcaggggcaaggccaatggacatatcaaattt
atcaagagccatttaaaaatctgaaaacaggaaaatatgcaagaatgaagggtgcccacactaatgatgtgaaacaattaac
agaggcagtacaaaaaatagccacagaaagcatagtaatatggggaaagactcctaaatttaaattacccatacaaaagga
aacatgggaagcatggtggacagagtattggcaagccacctggattcctgagtgggagtttgtcaatacccctcccttagtga
agttatggtaccagttagagaaagaacccataataggagcagaaactttctatgtagatggggcagccaatagggaaactaa
attaggaaaagcaggatatgtaactgacagaggaagacaaaaagttgtccccctaacggacacaacaaatcagaagactg
agttacaagcaattcatctagctttgcaggattcgggattagaagtaaacatagtgacagactcacaatatgcattgggaatcat
tcaagcacaaccagataagagtgaatcagagttagtcagtcaaataatagagcagttaataaaaaaggaaaaagtctacctg
gcatgggtaccagcacacaaaggaattggaggaaatgaacaagtagatgggttggtcagtgctggaatcaggaaagtacta
tttttagatggaatagataaggcccaagaagaacatgagaaatatcacagtaattggagagcaatggctagtgattttaaccta
ccacctgtagtagcaaaagaaatagtagccagctgtgataaatgtcagctaaaaggggaagccatgcatggacaagtaga
ctgtagcccaggaatatggcagctagattgtacacatttagaaggaaaagttatcttggtagcagttcatgtagccagtggatat
atagaagcagaagtaattccagcagagacagggcaagaaacagcatacttcctcttaaaattagcaggaagatggccagta
aaaacagtacatacagacaatggcagcaatttcaccagtactacagttaaggccgcctgttggtgggcggggatcaagcag
gaatttggcattccctacaatccccaaagtcaaggagtaatagaatctatgaataaagaattaaagaaaattataggacaggt
aagagatcaggctgaacatcttaagacagcagtacaaatggcagtattcatccacaattttaaaagaaaaggggggattggg
gggtacagtgcaggggaaagaatagtagacataatagcaacagacatacaaactaaagaattacaaaaacaaattacaaa
aattcaaaattttcgggtttattacagggacagcagagatccagtttggaaaggaccagcaaagctcctctggaaaggtgaag
gggcagtagtaatacaagataatagtgacataaaagtagtgccaagaagaaaagcaaagatcatcagggattatggaaaac
agatggcaggtgatgattgtgtggcaagtagacaggatgaggattaa

Vif

Atggaaaacagatggcaggtgatgattgtgtggcaagtagacaggatgaggattaacacatggaaaagattagtaaaacac
catatgtatatttcaaggaaagctaaggactggttttatagacatcactatgaaagtactaatccaaaaataagttcagaagtac
acatcccactaggggatgctaaattagtaataacaacatattggggtctgcatacaggagaaagagactggcatttgggtcag

ggagtctccatagaatggaggaaaaagagatatagcacacaagtagaccctgacctagcagaccaactaattcatctgcac
tattttgattgtttttcagaatctgctataagaaataccatattaggacgtatagttagtcctaggtgtgaatatcaagcaggacata
acaaggtaggatctctacagtacttggcactagcagcattaataaaaccaaaacagataaagccacctttgcctagtgttagg
aaactgacagaggacagatggaacaagccccagaagaccaagggccacagagggagccatacaatgaatggacacta
g

Vpr

atggaacaagccccagaagaccaagggccacagagggagccatacaatgaatggacactagagcttttagaggaactta
agagtgaagctgttagacattttcctaggatatggctccataacttaggacaacatatctatgaaacttacggggatacttgggc
aggagtggaagccataataagaattctgcaacaactgctgtttatccatttcagaattgggtgtcgacatagcagaataggcgt
tactcgacagaggagagcaagaaatggagccagtagatcc**tag**

Tat (x1)

atggagccagtagatcctagactagagccctggaagcatccaggaagtcagcctaaaactgcttgtaccaattgctattgtaa
aaagtgttgctttcattgccaagtttgtttcatgacaaaagccttaggcatctcctatggcaggaagaagcggagacagcgac
gaagagctcatcagaacagtcagactcatcaagcttctctatcaaagca

Rev (x1)

atggcaggaagaagcggagacagcgacgaagagctcatcagaacagtcagactcatcaagcttctctatcaaagca

Vpu

atgcaacctataatagtagcaatagtagcattagtagtagcaataataatagcaatagttgtgtggtccatagtaatcatagaata
taggaaaatattaagacaaagaaaaatagacaggttaattgatagactaatagaaagagcagaagacagtggcaatgagag
tgaaggagaagtatcagcacttgtggagatgggggtggaaatggggcaccatgctccttgggatattgatgatctg**tag**

Env

atgagagtgaaggagaagtatcagcacttgtggagatgggggtggaaatggggcaccatgctccttgggatattgatgatct
gtagtgctacagaaaaattgtgggtcacagtctattatggggtacctgtgtggaaggaagcaaccaccactctattttgtgcatc
agatgctaaagcatatgatacagaggtacataatgtttgggccacacatgcctgtgtacccacagaccccaacccacaaga
agtagtattggtaaatgtgacagaaaattttaacatgtggaaaaatgacatggtagaacagatgcatgaggatataatcagttta
tgggatcaaagcctaaagccatgtgtaaaattaaccccactctgtgttagtttaaagtgcactgatttgaagaatgatactaatac
caatagtagtagcgggagaatgataatggagaaaggagagataaaaaactgctctttcaatatcagcacaagcataagaga
taaggtgcagaaagaatatgcattcttttataaacttgatatagtaccaatagataataccagctataggttgataagttgtaacac
ctcagtcattacacaggcctgtccaaaggtatcctttgagccaattcccatacattattgtgccccggctggttttgcgattctaa
aatgtaataataagacgttcaatggaacaggaccatgtacaaatgtcagcacagtacaatgtacacatggaatcaggccagt
agtatcaactcaactgctgttaaatggcagtctagcagaagaagatgtagtaattagatctgccaatttcacagacaatgctaa
aaccataatagtacagctgaacacatctgtagaaattaattgtacaagacccaacaacaatacaagaaaaagtatccgtatcc
agaggggaccagggagagcatttgttacaataggaaaaataggaaatatgagacaagcacattgtaacattagtagagcaa
aatggaatgccactttaaaacagatagctagcaaattaagagaacaatttggaaataataaaacaataatctttaagcaatcct
caggaggggacccagaaattgtaacgcacagttttaattgtggaggggaatttttctactgtaattcaacacaactgtttaatagt
acttggtttaatagtacttggagtactgaagggtcaaataacactgaaggaagtgacacaatcacactcccatgcagaataaa

acaatttataaacatgtggcaggaagtaggaaaagcaatgtatgcccctcccatcagtggacaaattagatgttcatcaaatat
tactgggctgctattaacaagagatggtggtaataacaacaatgggtccgagatcttcagacctggaggaggcgatatgagg
gacaattggagaagtgaattatataaatataaagtagtaaaaattgaaccattaggagtagcacccaccaaggcaaagagaa
gagtggtgcagagagaaaaaagagcagtgggaataggagctttgttccttgggttcttgggagcagcaggaagcactatgg
gctgcacgtcaatgacgctgacggtacaggccagacaattattgtctgatatagtgcagcagcagaacaatttgctgagggc
tattgaggcgcaacagcatctgttgcaactcacagtctggggcatcaaacagctccaggcaagaatcctggctgtggaaag
atacctaaaggatcaacagctcctggggatttggggttgctctggaaaactcatttgcaccactgctgtgccttggaatgctagt
tggagtaataaatctctggaacagatttggaataacatgacctggatggagtgggacagagaaattaacaattacacaagctt
aatacactccttaattgaagaatcgcaaaaccagcaagaaaagaatgaacaagaattattggaattagataaatgggcaagtt
tgtggaattggtttaacataacaaattggctgtggtatataaaattattcataatgatagtaggaggcttggtaggtttaagaatagt
ttttgctgtactttctatagtgaatagagttaggcagggatattcaccattatcgtttcagacccacctcccaatcccgaggggac
ccgacaggcccgaaggaatagaagaagaaggtggagagagagacagagacagatccattcgattagtgaacggatcctt
agcacttatctgggacgatctgcggagcctgtgcctcttcagctaccaccgcttgagagacttactcttgattgtaacgaggatt
gtggaacttctgggacgcagggggtgggaagccctcaaatattggtggaatctcctacagtattggagtcaggaactaaaga
atagtgctgttaacttgctcaatgccacagccatagcagtagctgaggggacagatagggttatagaagtattacaagcagct
tatagagctattcgccacatacctagaagaataagacagggcttggaaaggattttgctataa

Tat (x2)

acccacctcccaatcccgaggggacccgacaggcccgaaggaatag

Rev (x2)

acccacctcccaatcccgaggggacccgacaggcccgaaggaatagaagaagaaggtggagagagagacagagaca
gatccattcgattagtgaacggatccttagcacttatctgggacgatctgcggagcctgtgcctcttcagctaccaccgcttga
gagacttactcttgattgtaacgaggattgtggaacttctgggacgcagggggtgggaagccctcaaatattggtggaatctcc
tacagtattggagtcaggaactaaagaatag

Nef

Atgggtggcaagtggtcaaaaagtagtgtgattggatggcctgctgtaagggaaagaatgagacgagctgagccagcagc
agatggggtgggagcagtatctcgagacctagaaaaacatggagcaatcacaagtagcaatacagcagctaacaatgctg
cttgtgcctggctagaagcacaagaggaggaagaggtgggttttccagtcacacctcaggtacctttaagaccaatgacttac
aaggcagctgtagatcttagccactttttaaaagaaaaggggggactggaagggctaattcactcccaaagaagacaagat
atccttgatctgtggatctaccacacacaaggctacttccctgattggcagaactacacaccagggccaggggtcagatatc
cactgacctttggatggtgctacaagctagtaccagttgagccagataaggtagaagaggccaataaaggagagaacacc
agcttgttacaccctgtgagcctgcatggaatggatgaccctgagagagaagtgttagagtggaggtttgacagccgcctag
catttcatcacgtggcccgagagctgcatccggagtacttcaagaactgc**tga**
